# Design, Synthesis, Cytotoxicity Assessment, and Molecular Docking of Novel Triazolopyrimidines as Potent Cyclin‐Dependent Kinase 4 Inhibitors

**DOI:** 10.1002/open.202500324

**Published:** 2025-11-02

**Authors:** Tariq Z. Abolibda, Sami A. Al‐Hussain, Basant Farag, Mohamed El‐Naggar, Magdi E. A. Zaki, Emad S. A. Alhazmi, Adel S. M. Almohammadi, Sobhi M. Gomha

**Affiliations:** ^1^ Department of Chemistry Faculty of Science Islamic University of Madinah Madinah 42351 Saudi; ^2^ Department of Chemistry Faculty of Science Imam Mohammad Ibn Saud Islamic University (IMSIU) Riyadh 11623 Saudi Arabia; ^3^ Department of Chemistry Faculty of Science Zagazig University Zagazig 44519 Egypt; ^4^ Department of Chemistry Pure and Applied Chemistry Group Faculty of Sciences University of Sharjah Sharjah 27272 UAE; ^5^ Medical Center Islamic University of Madinah Madinah 42351 Saudi Arabia

**Keywords:** 1,2,4‐triazolo[4,3‐a]pyrimidines, absorption, distribution, metabolism, excretion, and toxicity, antitumor activity, cyclin‐dependent kinase 4, hydrazonoyl halides, molecular docking

## Abstract

Cyclin‐dependent kinase 4 (CDK4) plays a pivotal role in cell cycle regulation and is a well‐established target in cancer therapy. Triazolopyrimidines, as bioactive heterocyclic compounds, represent a promising scaffold for the development of novel anticancer agents. Herein, a new series of 1,5‐dihydro‐[1,2,4]triazolo[4,3‐a]pyrimidine derivatives (**5a–g**) is synthesized via multistep reactions involving 6‐methyl‐4‐phenyl‐2‐thioxo‐1,2,3,4‐tetrahydropyrimidin‐5‐yl propionate and hydrazonoyl halides. Structural confirmation is achieved through infrared spectroscopy, ^1^H nuclear magnetic resonance, mass spectrometry, and elemental analysis, and further supported by alternative synthetic approaches. Molecular docking studies targeting the CDK4/cyclin D1 complex (PDB ID: 2W9Z) reveal favorable binding interactions, particularly for compounds **5c** and **5d**, with binding energies of −7.34 and −7.25 kcal/mol, respectively. In vitro cytotoxicity assays against HepG2 liver cancer cells show that compounds **5c, 5d,** and **5f** exhibit potent activity, with IC_50_ values of 4.38, 3.96, and 3.84 µM, respectively, comparable to doxorubicin (3.43 µM). A similar trend is observed in MCF‐7 breast cancer cells, where **5c**, **5d**, and **5f** again demonstrate strong antiproliferative effects with IC_50_ values of 4.12, 3.87, and 3.95 µM, respectively, close to doxorubicin (3.25 µM). The absorption, distribution, metabolism, excretion, and toxicity profile indicates excellent absorption, moderate distribution, low toxicity, and favorable drug‐likeness. These findings highlight the potential of the synthesized triazolopyrimidine derivatives as promising leads for CDK4‐targeted anticancer drug development.

## Graphical abstract

A novel series of 1,5‐dihydro‐[1,2,4]triazolo[4,3‐a]pyrimidines (5a–g) is synthesized and evaluated as potential CDK4 inhibitors. Compounds 5c and 5d exhibit strong cytotoxicity toward HepG2 and MCF‐7 cells with IC_5__0_ ≈ 1–2 µM, comparable to doxorubicin. Molecular docking reveals stable hydrogen‐bonding and π–H interactions with key residues Val14 and Glu144 in the CDK4–cyclin D1 complex.

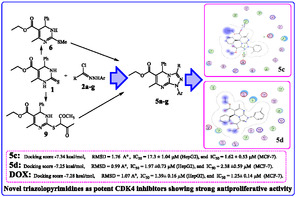

## 1 Introduction

Despite major advancements in understanding cancer biology—including signaling pathways, target identification, and drug development—cancer remains a leading cause of death globally, affecting both developed and developing nations alike.^[^ ^1^ ^]^ Characterized by uncontrolled cellular proliferation, cancer encompasses a spectrum of complex degenerative disorders.^[^ ^2^ ^]^ Central to this proliferation is the cell cycle, which is tightly regulated by cyclins and cyclin‐dependent kinases (CDKs); disruptions in this regulatory network are frequently linked to tumorigenesis.^[^ ^3^ ^,^ ^4^ ^]^ Cyclin D, through activation of CDK4 and CDK6, governs G1‐phase progression, and its aberrant overexpression is commonly observed across multiple cancers.^[^ ^5^, ^6^, ^7^ ^–^ ^8^ ^]^ As a result, the cyclin D1–CDK4/6 axis has emerged as a critical oncogenic pathway and an attractive therapeutic target. Selective inhibition of CDK4 is particularly compelling due to its pivotal role in cell cycle progression and its relatively favorable toxicity profile, making the development of CDK4‐targeted small molecules a high‐priority goal in anticancer drug discovery.^[^ ^9^, ^10^, ^11^, ^12^, ^13^, ^14^ ^–^ ^15^ ^]^

**Figure 1:**
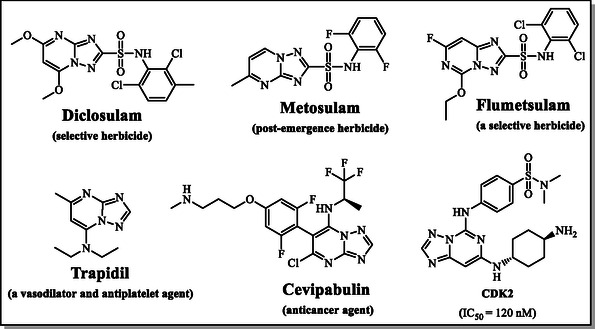
Structures of commercial triazolopyrimidines.

Nitrogen‐containing heterocycles, particularly triazoles and pyrimidines, have attracted considerable attention due to their broad pharmacological versatility, encompassing anticancer, anti‐inflammatory, antitubercular, antifungal, antibacterial, antiviral, antioxidant, antiproliferative, and herbicidal activities.^[^ ^16^, ^17^, ^18^, ^19^, ^20^, ^21^, ^22^ ^–^ ^23^ ^]^ Several such heterocycles—including Pyrithiobac‐sodium, Metosulam, and Flumetsulam—are utilized as commercial herbicides, while others like Trapidil and Essramycin have found pharmaceutical applications (**Figure** **1**).^[^ ^24^, ^25^, ^26^ ^–^ ^27^ ^]^ Among these, [1,2,4]^[^triazolo[1,5‐*a*]pyrimidines (TPs) have emerged as particularly promising scaffolds in medicinal chemistry due to their purine bioisosterism and multifaceted anticancer properties. These compounds inhibit CDKs by mimicking the purine ring and competitively occupying the ATP binding pocket, as supported by structure‐guided design, structure–activity relationship (SAR) analysis, and crystallographic validation, yielding potent and selective submicromolar CDK2 inhibitors.^[^ ^28^ ^,^ ^29^ ^]^ Moreover, TPs such as TTI‐237 (cevipabulin) exhibit microtubule‐targeting activity and remain effective in multidrug‐resistant cancer models, functioning through mechanisms distinct from traditional taxanes and vinca alkaloids.^[^ ^26^ ^]^ Related compounds like CNDR‐51,657 further demonstrate strong microtubule stabilization and favorable pharmacokinetic and brain‐penetration profiles, broadening their potential for neuro‐oncological applications.^[^ ^26^ ^,^ ^30^, ^31^, ^32^, ^33^, ^34^ ^]^ Recent reports also underscore renewed interest in selective CDK4 targeting and next‐generation scaffolds with improved pharmacokinetics, tolerability, and central nervous system penetration.^[^ ^34^, ^35^ ^–^ ^36^ ^]^

Complementing synthetic efforts, in silico tools such as molecular docking and absorption, distribution, metabolism, excretion, and toxicity (ADMET) prediction have become indispensable in modern drug discovery.^[^ ^37^ ^]^ In the present study, the crystal structure of CDK4 complexed with cyclin D1 (PDB ID: 2W9Z) was used for docking to evaluate the binding interactions of newly synthesized triazolopyrimidine derivatives (**5a–g, 6,** and **9**). Pharmacokinetic and toxicity profiles were predicted using pkCSM,^[^ ^38^ ^]^ and the compounds were subsequently assessed in vitro for their antiproliferative activity against HepG2 and MCF‐7 cancer cells, aiming to identify promising lead candidates for anticancer therapy.

Building on our previous efforts to synthesize bioactive heterocycles,^[^ ^39^, ^40^, ^41^, ^42^, ^43^, ^44^, ^45^, ^46^, ^47^, ^48^ ^–^ ^49^ ^]^ this study reports the design, synthesis, and evaluation of a novel series of 1,5‐dihydro‐[1,2,4]triazolo[4,3‐*a*]pyrimidine derivatives as potential CDK4 inhibitors. Molecular docking using the CDK4–cyclin D1 complex (PDB ID: 2W9Z) was employed to predict binding interactions, while in silico ADMET profiling (pkCSM) was conducted to assess pharmacokinetic properties and toxicity. The compounds were further evaluated in vitro for antiproliferative activity against HepG2 liver cancer cells to identify promising lead candidates for anticancer therapy.

## 2 Results and Discussion

### 2.1 Chemistry

**Scheme 1:**
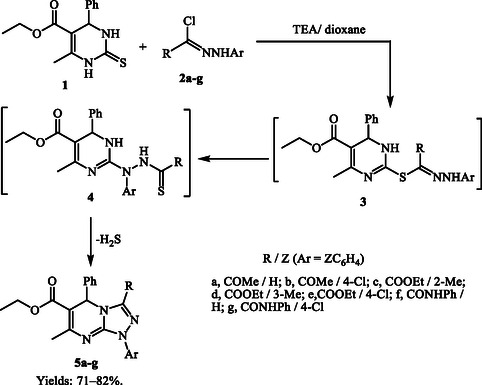
Synthesis of 1,5‐Dihydro‐[1,2,4]Triazolo[4,3‐*a*]pyrimidines **5a–g.**

6‐Methyl‐4‐phenyl‐2‐thioxo‐1,2,3,4‐tetrahydropyrimidin‐5‐yl propionate (**1**)^[^ ^50^ ^]^ was obtained and subsequently subjected to reactivity studies with hydrazonoyl halides. When **1** was treated with different hydrazonoyl halides **2a–g** ^[^ ^51^, ^52^ ^–^ ^53^ ^]^ in refluxing dioxane in the presence of triethylamine, a single product was isolated in each case, identified as compound **5** (**Scheme** **1**) through spectral analysis (infrared spectroscopy (IR), ^1^H nuclear magnetic resonance (NMR), mass spectrometry (MS)) and elemental composition (see Experimental). The proposed reaction mechanism (Scheme 1) suggests that the process initiates via S‐alkylation, forming unstable intermediates **3**, which then rearrange and cyclize in situ to form the final heterocyclic structures **5**.

**Scheme 2:**
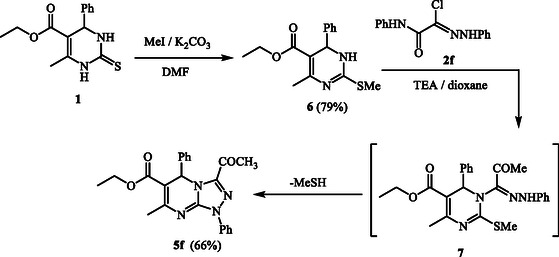
Alternate synthesis of **5f.**

Compounds **5** can also be synthesized through an alternative approach starting from the methylthio derivative **6**, which is prepared by reacting compound **1** with methyl iodide in DMF in the presence of anhydrous potassium carbonate. Subsequent reaction of **6** with hydrazonoyl halides in refluxing dioxane using triethylamine as a base result in the formation of the same products **5**. As a representative example, the reaction of **6** with hydrazonoyl chloride **2f** afforded a product identical in all respects (melting point, mixed melting point, and IR spectra) to compound **5a**. The reaction mechanism leading to **5f** from **6** and **2f** is depicted in **Scheme** **2**.

**Scheme 3:**
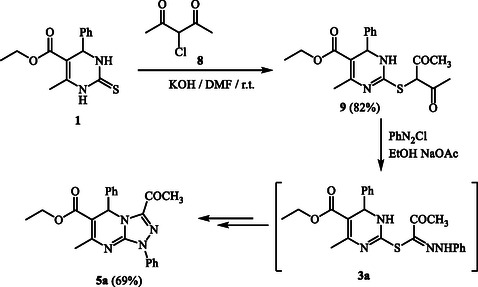
Alternative synthesis of **5a**.

The formation of compounds **5** is further supported by an alternative synthetic route to **5a**, which highlights the involvement of intermediates **3** and **4** (**Scheme** **3**). In this approach, compound **1** was reacted with the active chloromethylene compound 3‐chloropentane‐2,4‐dione (**8**) in DMF using KOH at room temperature, leading to the substitution product **9** (ethyl 2‐((2,4‐dioxopentan‐3‐yl)thio)‐4‐methyl‐6‐phenyl‐1,6‐dihydropyrimidine‐5‐carboxylate). The identity of **9** was confirmed through microanalysis and spectral techniques (IR, ^1^H NMR, MS), where the ^1^H NMR spectrum exhibited singlets near *δ* ≈ 2.48 and 4.24 ppm attributed to acetyl and methine protons.

Further transformation of **9** with benzenediazonium chloride in ethanol at 0–5 °C, in the presence of sodium acetate, yielded transient thiohydrazonate intermediates **3a**, which rearranged through a Smiles‐type mechanism to give **4a**, followed by cyclization with H_2_S release to produce **5a**. The product obtained matched compound **5a** (prepared from **1** or **6** and **2a**) in all analytical aspects (melting point, mixed melting point, IR), thereby confirming the intermediacy of **3** and **4**, which are rapidly consumed under the applied reaction conditions to form the final product **5**.

### 2.2 Molecular Docking Study

The crystal structure of CDK4 complexed with a D‐type cyclin was retrieved from the RCSB Protein Data Bank (PDB ID: 2W9Z).^[^ ^54^, ^55^, ^56^ ^–^ ^57^ ^]^ Molecular docking was performed to investigate the binding interactions between the target protein and the synthesized ligands. This computational method evaluates how ligands fit into the active site of a target protein and estimates the strength and nature of their interactions.^[^ ^58^ ^,^ ^59^ ^]^

**Figure 2:**
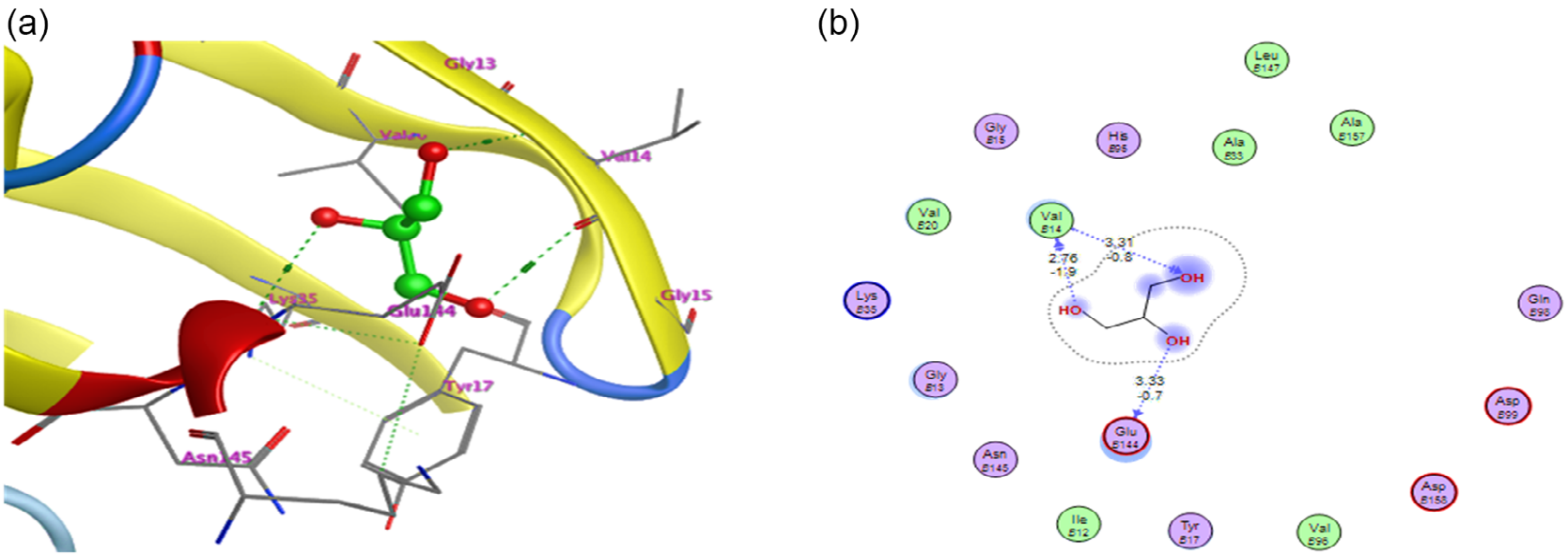
a) Three‐dimensional and b) two‐dimensional interactions of reference ligand with cyclin‐dependent kinase 4.

A co‐crystallized small‐molecule ligand appropriate for redocking validation cannot be found in the PDB structure 2W9Z. We used the following validation techniques to get around this restriction and guarantee the stability of our docking protocol: We chose a well‐known CDK4 inhibitor from a closely related PDB entry (such as 2W96), which has a reference ligand, by redocking it from a related structure, as presented in **Figure** **2**. Similar docking parameters were used in our work to redock this ligand to its native binding site. The resulting root mean square deviation (RMSD) of 1.90 Å between the docked and crystallographic poses confirmed the reliability of our docking technique.

**Literature Comparison:** The expected binding interactions between co‐crystallized ligand and CDK4 were in line with those documented for known CDK4 inhibitors, especially when it came to residues like Glu144 and Val14.

The following key findings were observed during the docking process: (i) The binding energies of the synthesized ligands were comparable to or exceeded that of the reference drugs doxorubicin and palbociclib, indicating stable complex formation. (ii) Low RMSD values suggested close alignment between the ligands and the protein's active site, particularly involving the functional groups. (iii) The interactions observed included hydrogen bond acceptors, *π*‐H, and hydrogen bond donors. (iv) The most significant amino acid residues involved in binding were Val14, Asp99, and Glu144 (**Figure** **3**). (v) The oxygen atoms, triazolo ring, and nitrogen sites were the primary interaction points within the ligands.

Figure 33D and 2D ligand interactions for novel synthesized compounds **5a–g**, **6**, and **9** in relation to the Dox. and Palbociclib within the binding site of 3W9Z.

**Figure:**
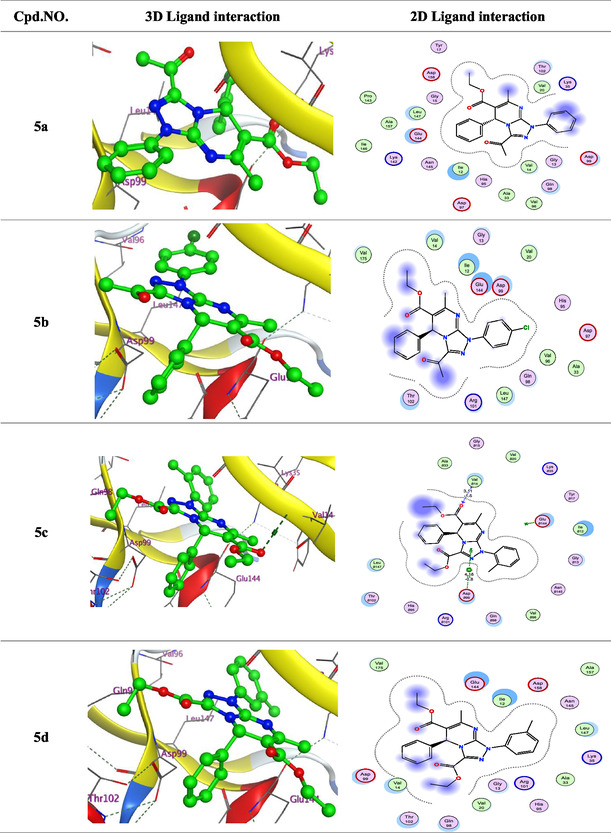

**Figure:**
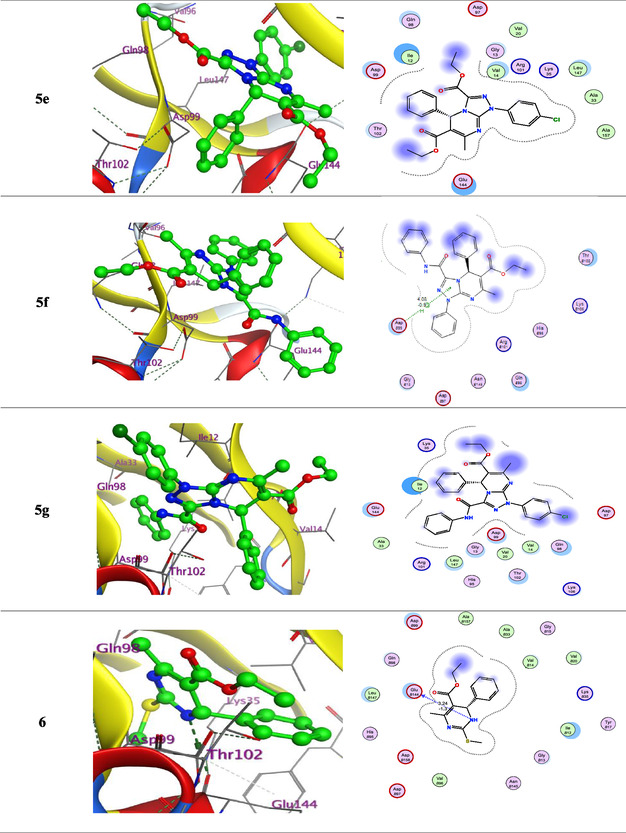

**Figure:**
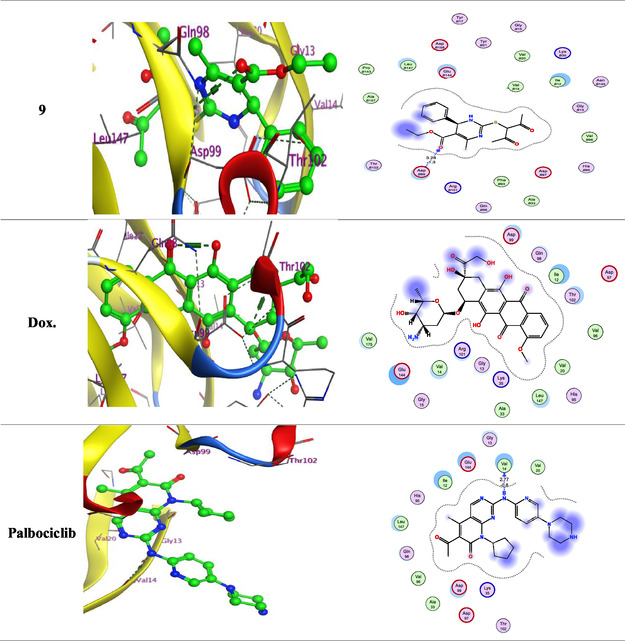

According to 3D interaction analysis, compounds **5c** and **9** formed hydrogen bond acceptor interactions between the Val14 residue and the carboxylate oxygen atom. Additionally, compounds **5c** and **5f** exhibited *π*‐H interactions between the triazolo ring and the critical Asp99 residue. Compound **6** formed a hydrogen bond donor interaction between the nitrogen atom of the pyrimidine ring and the Glu144 residue. Palbociclib formed a hydrogen bond donor interaction between the nitrogen atom of the amino group and the Val14 residue. These docking results suggest that the tested ligands could serve as promising inhibitors of cancer‐related CDK4 signaling pathways **Table** **1**.

**Table 1:** Docking score (kcal mol^–^^1^) and RMSD (A˚) of novel synthesized compounds **5a–g**, **6**, and **9** with the 2W9Z receptor.

Cpd. NO.	Docking score [kcal mol^–1^]	RMSD [A˚]
**5a**	−6.56	1.23
**5b**	−6.42	0.74
**5c**	−7.34	1.76
**5d**	−7.25	0.99
**5e**	−6.78	1.88
**5f**	−7.04	0.82
**5g**	−6.95	1.26
**6**	−6.41	1.13
**9**	−6.40	1.55
Dox.	−7.28	1.07
Palbociclib	−7.14	2.40

Based on each detail discussed before, **5c** will become a future lead candidate, and **5d**, **5f**, and **5g** are the highest docking scores, respectively, which are compatible with the biological activity and in silico ADMET profile of the most potent.

**Figure 4:**
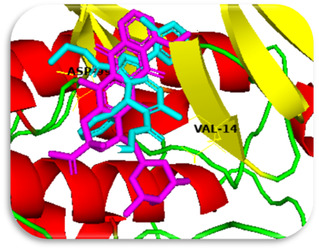
Superimposed binding conformation of derivative **5c** and DOX within the CDK4 active site. Ligands are color‐coded: **5c** in cyan and DOX in magenta.

Thus, novel 6‐methyl‐4‐phenyl‐pyrimidin‐5‐yl propionate derivatives could become a potential lead molecule against cancer, having better binding affinity, and controlling Cyclin D. We have created a superimposed binding conformation of the highest docking score derivative **5c** and doxorubicin within the CDK4 active site (**Figure** **4**).

The proposal that **5c** might function as a strong CDK4 inhibitor with added advantages is supported by the comparative visualization.

### 2.3 In Silico ADMET Pharmacokinetic Profile

A compound is generally classified as poorly absorbed when its intestinal absorption is below 30%.^[^ ^61^ ^]^ Based on ADMET prediction results, the most potent novel compounds **5a–g** exhibited favorable human intestinal absorption, ranging from 79.96% to 96.94%, indicating enhanced absorption characteristics for all tested molecules.

The volume of distribution at steady state (VDss) reflects the extent to which a drug distributes throughout the body relative to the plasma. A VDss greater than 2.81 L/kg is considered high, while a value below 0.71 L kg^–1^ is regarded as low.^[^ ^61^ ^]^ For the synthesized compounds, the predicted VDss (log L kg^–1^) ranged between 0.4 and 1.68, suggesting moderate distribution profiles.

Cytochrome P450 (CYP) enzymes, particularly CYP3A4 and CYP2D6, are key in the metabolism of numerous drugs and endogenous substances. The metabolic profiles of the tested derivatives, including potential CYP interactions, are summarized in **Table** **2**.

**Table 2:** Results of ADMET properties of the most potent novel synthetic compounds **5a–g**.

Cpd. NO.	Absorption	Distribution	Metabolism	Excretion	Toxicity
	Intestinal absorption [% Absorbed]	VDss [log L kg^–1^ −3.054 Numeric (log PS)		Renal OCT2 substrate [Yes/No]	Hepatotoxicity [Yes/No]
**5a**	91.86	1.77	CYP3A4 substrate and CYP2D6, CYP3A4 inhibitors	Yes	Yes
**5b**	84.31	0.4	CYP3A4 substrate and CYP2C19, CYP3A4 inhibitors	Yes	Yes
**5c**	96.94	1.68	CYP3A4 substrate and CYP3A4 inhibitor	No	Yes
**5d**	96.94	1.68		No	Yes
**5e**	95.48	1.61		No	Yes
**5f**	93.48	1.47	CYP3A4 substrate and CYP2C9, CYP3A4 inhibitors	No	Yes
**5g**	79.96	0.83	CYP2D6, CYP3A4 substrate and CYP1A2, CYP2C19, CYP2C9, CYP2D6 inhibitors	Yes	Yes

Renal excretion and clearance are heavily influenced by transporters such as the Renal Organic Cation Transporter 2 (OCT2). Among the evaluated compounds, only **5a**, **5b**, and **5g** were identified as potential OCT2 substrates, while the others were not.

Lastly, all derivatives **5a–g** exhibited hepatotoxicity in biological assays, consistent with the predicted ADMET outcomes.

Hence, these compounds will be more potent for future studies.

### 2.4 Cytotoxic Activity

The in vitro cytotoxic effects of the synthesized 1,5‐dihydro‐[1,2,4]triazolo[4,3‐a]pyrimidine derivatives **5a–g** were evaluated against HepG‐2 (liver carcinoma) and MCF‐7 (breast cancer) cancer cells using the crystal violet colorimetric assay, with doxorubicin serving as a reference drug. Dose–response curves were constructed to determine the IC_50_ values, representing the concentration required to inhibit 50% of cell viability. All tested compounds exhibited concentration‐dependent growth inhibition. Cytotoxicity results were expressed as mean IC_50_ values from three independent experiments. The cytotoxic activity results are summarized in **Table** **3**.

**Table 3:** IC_50_ values of the tested compounds against HepG‐2 and MCF‐7 cell lines.

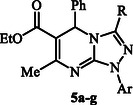				
Tested compounds	R	Ar	Antiproliferative activity IC_50_ ± SEM [µM]	
			HepG2	MCF‐7
**5a**	COMe	Ph	8.1 ± 0.82	9.7 ± 0.72
**5b**	COMe	4‐ClC_6_H_4_	17.3 ± 1.04	21.4 ± 0.91
**5c**	COOEt	2‐MeC_6_H_4_	1.35 ± 0.58	1.62 ± 0.53
**5d**	COOEt	3‐MeC_6_H_4_	1.97 ± 0.73	2.38 ± 0.59
**5e**	COOEt	4‐ClC_6_H_4_	7.37 ± 1.14	8.06 ± 1.02
**5f**	CONHPh	Ph	2.27 ± 0.93	2.74 ± 0.85
**5g**	CONHPh	4‐ClC_6_H_4_	5.32 ± 1.00	7.13 ± 1.04
Doxorubicin	–	–	1.39 ± 0.16	1.25 ± 0.14

### 2.5 SAR

**Figure 5:**
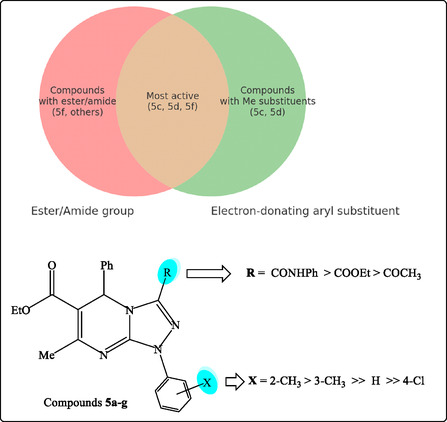
SAR of tested triazolopyrimidine derivatives **5a–g** against HepG‐2 and MCF‐7 cell lines.

The cytotoxic evaluation of the synthesized triazolopyrimidines against HepG2 and MCF‐7 cells revealed distinct activity patterns that provide valuable structure–activity insights (Table 3 and **Figure** **5**). Overall, the compounds demonstrated variable potency, with IC_50_ values ranging from low micromolar to >20 µM. Among them, derivatives **5c**, **5d**, and **5f** consistently exhibited the strongest activity across both cell lines, with values approaching those of the standard reference drug, doxorubicin. In particular, **5c** (COOEt, 2‐MeC_6_H_4_) was the most active compound, showing IC_50_ values of 1.35 µM against HepG2 and 1.62 µM against MCF‐7, which are nearly equivalent to doxorubicin (1.39 µM and 1.25 µM, respectively). Similarly, **5d** (COOEt, 3‐MeC_6_H_4_) displayed excellent activity (1.97 µM in HepG2; 2.38 µM in MCF‐7), while **5f** (CONHPh, Ph) was also highly potent (2.27 µM and 2.74 µM, respectively). These results confirm that both ester and amide functionalities significantly contribute to activity, likely by enhancing hydrogen bonding and improving cellular uptake.

When comparing the two cell lines, a subtle difference in sensitivity was observed. HepG2 cells appeared slightly more sensitive overall, as reflected by lower IC_50_ values for most derivatives. For instance, **5a** exhibited moderate inhibition in HepG2 (8.1 µM) but weaker activity in MCF‐7 (9.7 µM). Similarly, **5e** and **5g** showed stronger effects in HepG2 (7.37 and 5.32 µM, respectively) than in MCF‐7 (8.06 and 7.13 µM, respectively). In contrast, **5b** was consistently the least active compound in both models, with IC_50_ values of 17.3 µM in HepG2 and 21.4 µM in MCF‐7, highlighting that the combination of a ketone R group with a para‐chloro aryl substituent is unfavorable. These results suggest that while both liver and breast cancer cells respond to the triazolopyrimidines, liver cancer cells may be more susceptible, possibly reflecting differences in CDK4 dependence or drug uptake between the two cell types.

Taken together, the SAR analysis emphasizes that electron‐donating aryl substituents at ortho and meta positions (as in **5c** and **5d**) provide optimal activity, whereas electron‐withdrawing substituents such as para‐Cl decrease potency. Importantly, the consistent performance of **5c**, **5d**, and **5f** across both HepG2 and MCF‐7 models highlights their potential as lead structures.

Compared with doxorubicin, these compounds demonstrated comparable potency with improved scaffold novelty, indicating promise for further optimization. These findings provide a strong rationale for advancing **5c** and **5f** to broader screening, including additional cancer and normal cell lines, to validate spectrum and selectivity, as well as mechanistic studies to confirm CDK4 inhibition.

## 3 Experimental Section

### 3.1 Chemistry

Elemental (C, H, N) analyses were performed on a Perkin–Elmer 2400 analyzer. TLC was carried out on Merck silica gel GF254 plates, and melting points were measured using an Electrothermal Gallenkamp apparatus. IR spectra (KBr discs) were obtained with a Pye‐Unicam SP300 instrument. ^1^H (500 MHz) and ^13^C (125 MHz) NMR spectra were recorded on a Jeol‐500 spectrometer in DMSO‐d_6_. Mass spectra were acquired on a Thermo Scientific GC/MS ISQ or an Agilent LC‐MSD IQ Infinity II 1260 system.

### 3.2 Synthesis of ethyl 1‐aryl‐7‐methyl‐5‐phenyl‐3‐substituted‐1,5‐dihydro‐[1,2,4]triazolo[4,3‐a]pyrimidine‐6‐carboxylate 5a–g

*General procedure:* To a mixture of equimolar amounts of 6‐methyl‐4‐phenyl‐2‐thioxo‐1,2,3,4‐tetrahydropyrimidin‐5‐yl propionate **3** (0.276 g, 1 mmol) and the appropriate hydrazonoyl halides **2a–g** (1 mmol) in dioxane (50 mL) was added TEA (0.14 mL, 1 mmol) at room temperature. The reaction mixture was refluxed till all hydrogen sulfide gas ceased to evolve (6–10 h, monitored by TLC). The solvent was then evaporated, and the residue was triturated with MeOH. The precipitate formed was filtered and recrystallized from the proper solvent to give the products **5a–g**, respectively.

### 3.3 Ethyl 3‐acetyl‐7‐methyl‐1,5‐diphenyl‐1,5‐dihydro‐[1,2,4]Triazolo[4,3‐a]pyrimidine‐6‐carboxylate (5a)

Yellowish‐white solid, mp 238–240 °C (DMF); yield 71%; IR (KBr): υ = 3054, 2927 (C—H), 1729, 1697 (2 C=O), 1608 (C=N) cm^−1^; ^1^H NMR (300 MHz, DMSO‐*d*_*6*_): *δ* = 1.07–1.09 (t, 3H, CH_3_CH_2_), 2.26 (s, 3H, CH_3_), 2.39 (s, 3H, CH_3_), 3.95–3.98 (q, 2H, CH_3_ CH_2_), 5.14 (s, 1H, pyrimidine‐H4), 7.18–7.97 (m, 10H, Ar‐H) ppm; ^13^C NMR (DMSO‐*d*_6_, 125 MHz) *δ* = 14.5, 17.7, 21.8 (CH_3_), 54.6 (CH), 60.1 (CH_2_), 123.0, 126.7, 126.9, 128.1, 129.0, 130.0, 134.6, 136.4, 144.0, 145.5, 151.3, 155.6 (Ar–C and C=N), 167.5, 193.8 (C=O) ppm; MS (70 eV): *m*/*z* = 402 (M^+^, 38). Anal. Calcd. for C_23_H_22_N_4_O_3_ (402.45): C, 68.64; H, 5.51; N, 13.92. Found: C, 68.55; H, 5.40; N, 13.75%.

### 3.4 Ethyl 3‐acetyl‐1‐(4‐chlorophenyl)‐7‐methyl‐5‐phenyl‐1,5‐dihydro‐[1,2,4]triazolo[4,3‐a]pyrimidine‐6‐carboxylate (5b)

Yellowish‐white solid, mp 267–269 °C (DMF); yield 73%; IR (KBr): υ = 3075, 2922 (C—H), 1728, 1692 (2 C=O), 1611 (C=N) cm^−1^; ^1^H NMR (300 MHz, DMSO‐*d*_*6*_): *δ* = 1.06–1.12 (t, 3H, CH_3_CH_2_), 2.27 (s, 3H, CH_3_), 2.39 (s, 3H, CH_3_), 3.97–3.99 (q, 2H, CH_3_ CH_2_), 5.15 (s, 1H, pyrimidine‐H4), 7.25–7.75 (m, 9H, Ar‐H) ppm; MS (70 eV): *m*/*z* = 438 (M^+^ + 2, 10), 436 (M^+^, 35). Anal. Calcd. for C_23_H_21_ClN_4_O_3_ (436.90): C, 63.23; H, 4.85; N, 12.82. Found: C, 63.06; H, 4.77; N, 12.69%.

### 3.5 Diethyl 7‐methyl‐5‐phenyl‐1‐(*o*‐tolyl)‐1,5‐dihydro‐[1,2,4]triazolo[4,3‐*a*]pyrimidine‐3,6‐dicarboxylate (5c)

Yellowish‐white solid, mp 223–235 °C (DMF/EtOH); yield 70%; IR (KBr): *υ* = 3036, 2917 (C—H), 1731, 1727, 1695 (3 C=O), 1607 (C=N) cm^−1^; ^1^H NMR (300 MHz, DMSO‐*d*_*6*_): *δ* = 1.05–1.10 (2t, 6H, 2CH_3_CH_2_), 2.10 (s, 3H, CH_3_), 2.26 (s, 3H, CH_3_), 3.97–4.05 (2q, 4H, 2CH_3_ CH_2_), 5.14 (s, 1H, pyrimidine‐H4), 6.85–7.49 (m, 9H, Ar‐H) ppm; ^13^C NMR (DMSO‐*d*_6_, 125 MHz) *δ* = 14.4, 17.2, 17.8, 21.2 (CH_3_), 54.6 (CH), 60.2 (CH_2_), 123.0, 126.2, 127.0, 127.4, 128.5, 129.3, 129.7, 130.4, 133.9, 135.3, 143.2, 145.8, 151.7, 155.6 (Ar–C and C=N), 167.4, 193.9 (C=O) ppm; MS (70 eV): *m*/*z* = 446 (M^+^, 79). Anal. Calcd. for C_25_H_26_N_4_O_4_ (446.51): C, 67.25; H, 5.87; N, 12.55. Found: C, 67.05; H, 5.72; N, 12.41%.

### 3.6 Diethyl 7‐methyl‐5‐phenyl‐1‐(*m*‐tolyl)–1,5‐dihydro‐[1,2,4]triazolo[4,3‐*a*]pyrimidine‐3,6‐dicarboxylate (5d)

Yellow solid, mp 179–181 °C (EtOH); yield 69%; IR (KBr): *υ* = 3055, 2929 (C—H), 1731, 1724, 1690 (3 C=O), 1604 (C=N) cm^−1^; ^1^H NMR (300 MHz, DMSO‐*d*_*6*_): *δ* = 1.05–1.09 (t, 2H, CH_3_CH_2_), 1.35–1.41 (t, 2H, CH_3_CH_2_), 2.21 (s, 3H, CH_3_), 2.38 (s, 3H, CH_3_), 3.97–3.99 (q, 2H, CH_3_ CH_2_), 4.27–4.30 (q, 2H, CH_3_ CH_2_), 5.15 (s, 1H, pyrimidine‐H4), 7.04–7.69 (m, 9H, Ar‐H) ppm; MS (70 eV): *m*/*z* = 446 (M^+^, 70). Anal. Calcd. for C_25_H_26_N_4_O_4_ (446.51): C, 67.25; H, 5.87; N, 12.55. Found: C, 67.17; H, 5.69; N, 12.47%.

### 3.7 Diethyl 1‐(4‐chlorophenyl)‐7‐methyl‐5‐phenyl‐1,5‐dihydro‐[1,2,4]triazolo[4,3‐a]pyrimidine‐3,6‐dicarboxylate (5e)

Yellowish‐white solid, mp 192–194 °C (EtOH); yield 71%; IR (KBr): υ = 3060, 2935 (C—H), 1734, 1729, 1691 (3 C=O), 1612 (C=N) cm^−1^; ^1^H NMR (300 MHz, DMSO‐*d*_*6*_): *δ* = 1.06–1.13 (t, 2H, CH_3_CH_2_), 1.40–1.48 (t, 2H, CH_3_CH_2_), 2.35 (s, 3H, CH_3_), 3.97–3.98 (q, 2H, CH_3_ CH_2_), 4.30–4.32 (q, 2H, CH_3_ CH_2_), 5.15 (s, 1H, pyrimidine‐H4), 7.19–7.60 (m, 9H, Ar‐H) ppm; MS (70 eV): *m*/*z* = 468 (M^+^ + 2, 7), 466 (M^+^, 24). Anal. Calcd. for C_24_H_23_ClN_4_O_4_ (466.92): C, 61.74; H, 4.97; N, 12.00. Found: C, 61.63; H, 4.85; N, 11.83%.

### 3.8 Ethyl 7‐methyl‐1,5‐diphenyl‐3‐(phenylcarbamoyl)–1,5‐dihydro‐[1,2,4]triazolo[4,3‐a]pyrimidine‐6‐carboxylate (5f)

Yellow solid, mp 269–271 °C (DMF); yield 75%; IR (KBr): υ = 3054, 2927 (C—H), 1729, 1657 (2 C=O), 1608 (C=N) cm^−1^; ^1^H NMR (300 MHz, DMSO‐*d*_*6*_): *δ* = 1.07–1.10 (t, 3H, CH_3_CH_2_), 2.26 (s, 3H, CH_3_), 3.96–4.03 (q, 2H, CH_3_ CH_2_), 5.14 (s, 1H, pyrimidine‐H4), 6.86–7.80 (m, 15H, Ar‐H), 10.18 (s, D_2_O exchangeable, 1H, NH) ppm; ^13^C NMR (DMSO‐*d*_6_, 125 MHz) *δ* = 14.3, 21.2 (CH_3_), 54.6 (CH), 60.0 (CH_2_), 121.5, 122.8, 124.5, 126.4, 127.0, 127.7, 128.3, 128.7, 129.3, 130.0, 132.7, 134.8, 144.1, 145.5, 151.3, 155.9 (Ar–C and C=N), 167.2, 169.8 (C=O) ppm; MS (70 eV): *m*/*z* = 479 (M^+^, 100). Anal. Calcd. for C_28_H_25_N_5_O_3_ (479.54): C, 70.13; H, 5.26; N, 14.60. Found: C, 70.04; H, 5.34; N, 14.53%.

### 3.9 Ethyl 1‐(4‐chlorophenyl)‐7‐methyl‐5‐phenyl‐3‐(phenylcarbamoyl)–1,5‐dihydro‐[1,2,4]triazolo[4,3‐a]pyrimidine‐6‐carboxylate (5g)

Yellowish‐white solid, mp 287–289 °C (DMF); yield 77%; IR (KBr): υ = 3047, 2925 (C—H), 1729, 1663 (2 C=O), 1604 (C=N) cm^−1^; ^1^H NMR (300 MHz, DMSO‐*d*_*6*_): *δ* = 1.07–1.10 (t, 3H, CH_3_CH_2_), 2.39 (s, 3H, CH_3_), 3.96–3.98 (q, 2H, CH_3_ CH_2_), 5.15 (s, 1H, pyrimidine‐H4), 7.12–7.78 (m, 14H, Ar‐H), 10.19 (s, D_2_O exchangeable, 1H, NH) ppm; MS (70 eV): *m*/*z* = 515 (M^+^ + 2, 17), 513 (M^+^, 55). Anal. Calcd. for C_28_H_24_ClN_5_O_3_ (513.98): C, 65.43; H, 4.71; N, 13.63. Found: C, 65.35; H, 4.58; N, 13.51%.

### 3.10 Synthesis of ethyl 4‐methyl‐2‐(methylthio)‐6‐phenyl‐1,6‐dihydropyrimidine‐5‐carboxylate (6)

Anhydrous K_2_CO_3_ (2.07 g, 15 mmol) and methyl iodide (1.42 g, 10 mmol) were added onto thione **1** (2.14 g, 10 mmol) in 20 mL *DMF,* and the reaction mixture was stirred at room temperature for 5h then poured onto ice–water mixture. The solid formed was filtered, washed with water, dried and crystallized from *DMF* to give compound **6** as white crystals in 79% yield, mp 254–256 °C; IR (KBr): υ = 3049, 2923 (C—H), 1729 (C=O), 1600 (C=N) cm^−1^; ^1^H NMR (300 MHz, DMSO‐*d*_*6*_): *δ* = 1.05–1.08 (t, 3H, CH_3_CH_2_), 2.16 (s, 3H, CH_3_), 2.57 (s, 3H, SCH_3_), 3.95–3.98 (q, 2H, CH_3_ CH_2_), 5.15 (s, 1H, pyrimidine‐H4), 7.18–7.32 (m, 5H, Ar‐H), 9.62 (s, D_2_O exchangeable, 1H, NH) ppm; ^13^C NMR (DMSO‐*d*_6_, 125 MHz) *δ* = 14.5, 16.7, 21.9 (CH_3_), 54.6 (CH), 60.1 (CH_2_), 121.3, 126.9, 128.2, 129.1, 144.0, 145.6 (Ar–C and C=N), 165.6, 167.8 (C=O) ppm; MS (70 eV): *m*/*z* = 290 (M^+^, 63). Anal. Calcd. for C_15_H_18_N_2_O_2_S (290.38): C, 62.04; H, 6.25; N, 9.65. Found: C, 62.13; H, 6.21; N, 9.45%.

### 3.11 Alternate Synthesis of 5f

Triethylamine (0.14 mL, 1 mmol) was added to a mixture of equimolar amounts of the methylthio derivative **6** (0.290 g, 1 mmol) and 2‐oxo‐N‐phenyl‐2‐(phenylamino) acetohydrazonoyl chloride (**2f**) (0.273 g, 1 mmol) in 50 mL of dioxane at room temperature. The reaction mixture was refluxed till all methanethiol ceased to evolve (10–12 h, monitored by TLC). The solvent was evaporated, and the residue was triturated with MeOH. The solid that formed was filtered and recrystallized from DMF to give compound **5f** (66% yield) which was identical in all respects (m.p., mixed m.p., and IR spectra) with that obtained from reaction of **1** with **2f.**

### 3.12 Alternate Synthesis of 5a

*Synthesis of ethyl 2‐((2,4‐dioxopentan‐3‐yl)thio)‐4‐methyl‐6‐phenyl‐1,6‐dihydropyrimidine‐5‐carboxylate (9)*: An aqueous solution of KOH (1 mL, 75%) was added onto thione **1** (2.76 g, 10 mmol) in 50 mL of EtOH, and the mixture was warmed for 10 min. in a water bath at 80 °C and cooled. To the resulting clear solution was added 3‐chloropentane‐2,4‐dione **8** (1.34g, 10 mmol) dropwise while stirring the reaction mixture. After complete addition, the reaction mixture was stirred for further 24 h at room temperature. The solid that precipitated was filtered off, washed with water, dried and crystallized from the proper solvent to give **9** as white solid, mp 166–168 °C (EtOH); yield 82%; IR (KBr): υ = 3058, 2925 (C—H), 1730, 1704 (2 C=O), 1603 (C=N) cm^−1^; ^1^H NMR (300 MHz, DMSO‐*d*_*6*_): *δ* = 1.05–1.08 (t, 3H, CH_3_CH_2_), 2.26 (s, 3H, CH_3_), 2.42 (s, 3H, CH_3_), 3.95–3.99 (q, 2H, CH_3_ CH_2_), 4.29 (s, 1H, CH), 5.15 (s, 1H, pyrimidine‐H4), 7.18–7.31 (m, 5H, Ar‐H), 9.61 (s, D_2_O exchangeable, 1H, NH) ppm; ^13^C NMR (DMSO‐*d*_6_, 125 MHz) *δ* = 14.5, 17.7, 23.6 (CH_3_), 54.6 (CH), 60.1 (CH_2_), 64.5 (CH), 126.9, 128.1, 129.0, 138.3, 144.0, 145.5, 154.2 (Ar–C and C=N), 167.6, 194.8 (C=O) ppm; MS (70 eV): *m*/*z* = 374 (M^+^, 63). Anal. Calcd. for C_19_H_22_N_2_O_4_S (374.46): C, 60.94; H, 5.92; N, 7.48. Found: C, 60.75; H, 5.81; N, 7.37%.

### 3.13 Coupling of Compound 9 Benzenediazonium Chloride

To a stirred solution of the corresponding compound **9** (0.374g, 1mmol) in ethanol (20mL), sodium acetate trihydrate (0.138g, 1mmol) was added, and the mixture was cooled to 0–5 °C in an ice bath. A freshly prepared cold solution of benzenediazonium chloride—obtained by diazotizing aniline (0.093g, 1mmol) in 6M HCl (1mL) with sodium nitrite (0.07g, 1mmol) in water (2mL)—was added dropwise. After complete addition, the mixture was stirred for 30min at 0–5 °C. The resulting solid was collected by filtration, washed with water, dried, and recrystallized from DMF to yield compound **5a** (69% yield), which matched the product obtained from the reaction of **1** with **2a** in melting point, mixed melting point, and IR spectra.

### 3.14 Docking Study

*Ligand Preparation*: The novel synthesized compounds were sketched using ChemDraw Professional 22.0. After structural optimization and energy minimization, the ligands were saved in MDB format to ensure compatibility with the molecular docking software.^[^ ^62^ ^]^

### 3.15 Protein Preparation

The X‐ray crystal structure of the enzyme (PDB ID: 2W9Z) was downloaded in PDB format. For docking analysis, the protein was processed through several steps. First, the relevant chains were preserved, while crystallographic water molecules were removed. Hydrogen atoms were then added based on standard geometries, and broken bonds were repaired to restore structural integrity.^[^ ^63^ ^]^

**Figure 6:**
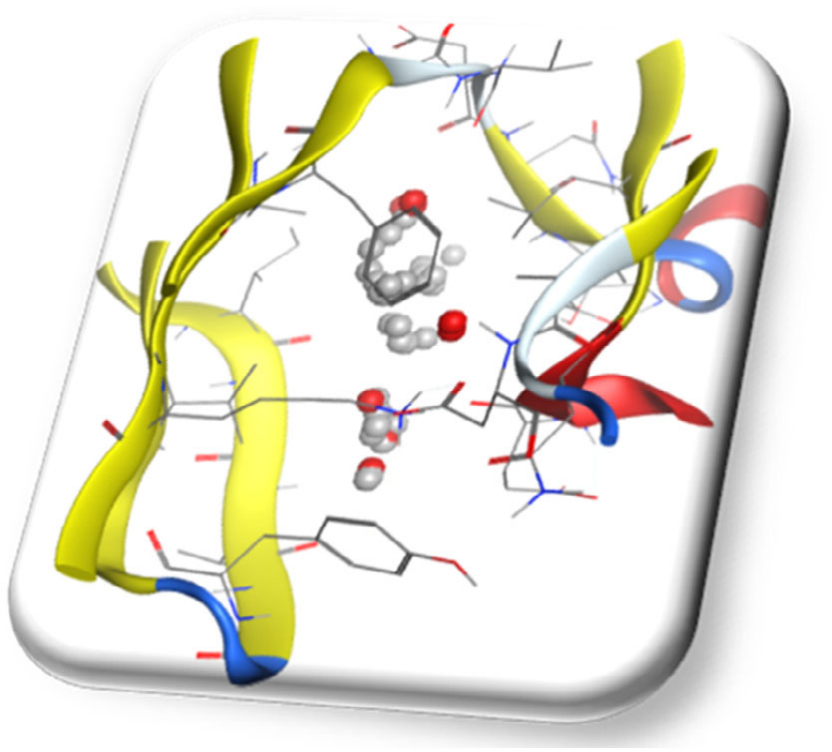
Prepared target protein (the co‐crystal structure of 2W9Z).

Subsequently, energy minimization was carried out using the MMFF94x force field until a RMSD gradient of 0.1 kcal mol^−1^ Å^−1^ was achieved, ensuring the stability of the protein structure.^[^ ^64^ ^]^ To identify the potential binding region, dummy atoms were generated using the Alpha Site Finder tool. The binding pocket of the protein was then marked and saved in MOE format, which was later used for simulating ligand–enzyme interactions at the active site. The final prepared protein structure is illustrated in **Figure** **6**.

### 3.16 Molecular Docking Analysis

Following the completion of molecular docking calculations, multiple docking poses were generated for each ligand conformer. These conformers were then ranked according to their docking scores, which reflect the predicted binding affinity. Both 2D and 3D visualizations were used to examine the interactions between the ligands and key amino acid residues within the active site. In particular: Residue Labels: The names and sequence positions of every interacting amino acid residue are now visibly shown. Different types of interactions are clearly marked with color codes and standardized symbols. Distance Indicators: To emphasize the spatial interactions between ligand and receptor atoms, key interaction distances are now shown in 2D.

The nature of these interactions was carefully analyzed to understand the binding orientation and stability of each compound. All docking procedures and scoring evaluations were performed in accordance with established protocols.^[^ ^65^ ^]^

### 3.17 In Silico ADMET Pharmacokinetic Profile

The pharmacokinetic and toxicological properties of the most promising synthesized compounds were predicted using the pkCSM web server.^[^ ^66^ ^]^ This publicly accessible tool (http://structure.bioc.cam.ac.uk/pkcsm, accessed on 18 May 2025) was utilized to assess ADMET (Absorption, Distribution, Metabolism, Excretion, and Toxicity) characteristics, offering valuable insight into the drug‐likeness and safety profile of each compound.^[^ ^67^, ^68^, ^69^, ^70^ ^–^ ^71^ ^]^

### 3.18 Evaluation of Antitumor Activity via Viability Assay

*Cell Culture* The human hepatocellular carcinoma cell line HepG2 (RRID:CVCL_0027) and the human breast adenocarcinoma cell line MCF‐7 (RRID:CVCL_0031) were obtained from the American Type Culture Collection (ATCC, Manassas, VA, USA). Cells were maintained in Dulbecco's Modified Eagle Medium (DMEM; Gibco, Thermo Fisher Scientific, USA) supplemented with 10% heat‐inactivated fetal bovine serum (FBS; Gibco) and 1% penicillin–streptomycin solution (100 U/mL penicillin, 100 µg/mL streptomycin). Cultures were incubated at 37 °C in a humidified atmosphere containing 5% CO_2_ and sub‐cultured routinely.

### 3.19 Viability Assay

Cytotoxic activity of the test compounds was evaluated according to the method of Gangadevi and Muthumary.^[^ ^72^ ^]^ Briefly, cells were seeded at a density of 1 × 10^4^ cells/well in 96‐well plates and incubated for 24 h. After washing with phosphate‐buffered saline (PBS, 0.01 M, pH 7.2), cells were treated with 100 µL of serial dilutions of the test compounds in fresh medium and incubated for an additional 24 h. Untreated cells served as negative controls, while 5‐fluorouracil, imatinib, and doxorubicin were used as positive controls.

Following treatment, cells were examined under an inverted microscope, stained with crystal violet, lysed with 33% glacial acetic acid, and absorbance was measured at 590 nm using a microplate reader (SunRise, TECAN, USA). Untreated wells were considered as 100% viability. The percentage of viable cells was calculated using the equation:

(1) $$\mathrm{Viability}(\%)=\left[1-\frac{OD_{t}}{OD_{c}}\right]\times 100$$

where *OD*_t_ is the optical density of treated wells and *OD*_c_ that of control wells. Dose–response curves were plotted, and IC_50_ values (concentration required to inhibit 50% of cell viability) were determined for each compound.

## 4 Conclusion

In this study, a novel series of 1,5‐dihydro‐[1,2,4]triazolo[4,3‐*a*]pyrimidine derivatives (**5a–g**) was successfully synthesized and structurally confirmed by spectral and elemental analyses. Among them, compounds **5c**, **5d**, and **5f** showed significant antiproliferative activity against HepG2 and MCF‐7 cancer cell lines, with IC_50_ values comparable to doxorubicin, highlighting their broad anticancer potential. Molecular docking studies revealed favorable binding of **5c** within the CDK4 active site, consistent with its in vitro potency, while ADMET predictions indicated promising pharmacokinetic properties. Although apoptosis assays, cell cycle analysis, kinase selectivity profiling (e.g., against CDK2/CDK6), and metabolic stability tests were not performed in this work, these are important next steps to validate the proposed mechanism and therapeutic relevance. The findings collectively suggest that triazolopyrimidine scaffolds, particularly **5c** and **5d**, represent promising leads for further optimization toward selective CDK4‐targeted anticancer agents.

## Conflict of Interest

The authors declare no conflict of interest.

## Supporting information

Supplementary Material

## Data Availability

The data that support the findings of this study are available from the corresponding author upon reasonable request.

## References

[1] K. B. Lokhande , S. Nagar , K. V. Swamy , Sci. Rep. 2019, 9, 1778. 30741976 10.1038/s41598-018-38332-6 PMC6370771

[2] M. H. El‐Shershaby , A. Ghiaty , A. H. Bayoumi , H. E. Ahmed , M. S. El‐Zoghbi , K. El‐Adl , H. S. Abulkhair , New J. Chem. 2021, 45, 11136.

[3] S. Man , I. O. Ellis , M. Sibbering , R. W. Blamey , J. D. Brook , Cancer Res. 1996, 56, 5484. 8968105

[4] F. Hommura , H. Dosaka‐Akita , I. Kinoshita , T. Mishina , H. Hiroumi , S. Ogura , H. Katoh , Y. Kawakami , Br. J. Cancer 1999, 81, 696. 10574258 10.1038/sj.bjc.6690750 PMC2362891

[5] D. Fry , M. Garrett , Curr. Opin. Oncol. Endocr. Metab. Investig. 2000, 2, 2.

[6] M. D. Garrett , Fattaey , A. CDK Inhibition and Cancer Therapy. Curr. Opin. Genet. Dev. 1999, 9, 104. 10072351 10.1016/s0959-437x(99)80015-x

[7] T. Motokura , T. Bloom , H. G. Kim , H. Jüppner , J. V. Ruderman , H. M. Kronenberg , A. Arnold , Nature 1991, 350, 512. 1826542 10.1038/350512a0

[8] M. Hall , G. Peters , Adv. Cancer Res. 1996, 68, 67. 8712071 10.1016/s0065-230x(08)60352-8

[9] D. A. Belchis , C. D. Gocke , J. Geradts , Pediatr. Pathol. Mol. Med. 2000, 19, 377.

[10] S. M. Gomha , S. M. Riyadh , A. A. A. El‐Sayed , A. M. Abdallah , M. E. A. Zaki , A. Alrehaily , H. M. Elbadawy , A. A. Al‐Shahri , S. R. Alsenani , A. M. Hussein . Inorg. Chem. Commun. 2024, 169, 113128.

[11] K. Gurushankar , H. Rimac , V. Potemkin , M. Grishina , J. Mol. Struct. 2021, 1230, 129925.

[12] G. Kumar , A. Varshney , N. K. Lohani , J. Exp. Biol. 2013, 1, 2S.

[13] S. Nussbaumer , P. Bonnabry , J.‐L. Veuthey , S. A. of Fleury‐Souverain , Anticancer Drugs: A Review. Talanta 2011, 85, 2265. 21962644 10.1016/j.talanta.2011.08.034

[14] J. G. Lombardino , J. A. Lowe , Nat. Rev. Drug Discov. 2004, 3, 853. 15459676 10.1038/nrd1523

[15] M. Peyressatre , C. Prével , M. Pellerano , M. C. Morris , Cancers Basel 2015, 7, 179. 25625291 10.3390/cancers7010179 PMC4381256

[16] N. Kerru , L. Gummidi , S. Maddila , K. K. Gangu , S. B. Jonnalagadda , Molecules 2020, 25, 1909. 32326131 10.3390/molecules25081909 PMC7221918

[17] S. Wang , X. H. Yuan , S. Q. Wang , W. Zhao , X. B. Chen , B. Yu , Eur. J. Med. Chem. 2021, 214, 113218. 33540357 10.1016/j.ejmech.2021.113218

[18] J. Lin , S. Zhou , J. X. Xu , W. Q. Yao , G. F. Hao , Y. T. D. Li , J. Agric. Food Chem. 2020, 68, 6792. 32442369 10.1021/acs.jafc.9b07887

[19] R. B. Aher , D. Sarkar , Modeling and Two‐Fold Classification of 1,2,4‐Triazole Derivatives for Antitubercular Potency Against the Dormant Stage of *Mycobacterium Tuberculosis* Mol. Divers. 2022, 26, 1227. 34347229 10.1007/s11030-021-10254-y

[20] R. R. Gajjala , R. R. Chinta , V. S. R. Gopireddy , S. Poola , S. K. Balam , V. Chintha , V. R. Pasupuleti , V. K. R. Avula , S. Vallela , G. V. Zyryanov , Cirandur , S.R. Ethyl‐4‐(Aryl)‐6‐Methyl‐2‐(Oxo/Thio)‐3,4‐Dihydro‐1H‐Pyrimidine‐5‐Carboxylates: Synthesis and Antioxidant Activity. Bioorg. Chem. 2022, 129, 106205. 10.1016/j.bioorg.2022.106205 36265354

[21] H. Rashid , M. A. U. Martines , A. P. Duarte , J. Jorge , S. Rasool , R. Muhammad , N. Ahmad , Umar , M.N. Syntheses and Anti‐Inflammatory Activities of Pyrimidines: A Review. RSC Adv. 2021, 11, 6060. 10.1039/d0ra10657g PMC8694831 35423143

[22] M. Devi , S. Jaiswal , S. Jain , N. Kaur , Dwivedi , J. Synthetic and Biological Attributes of Pyrimidine Derivatives: A Recent Update. Curr. Org. Synth. 2021, 18, 790. 10.2174/1570179418666210706152515 34886770

[23] J. Luo , H. Nie , L. He , A. Zhao , T. N. Wang , J. Mol. Struct. 2024, 1300, 137246.

[24] A. A. Mandour , I. F. Nassar , M. T. Abdel Aal , M. A. E. Shahin , W. A. El‐Sayed , M. Hegazy , A. M. Yehia , A. Ismail , M. Hagras , E. B. Elkaeed , H. M. Refaat , N. S. M. Ismail , J. Enzyme Inhib. Med. Chem. 2022, 37, 1957. 35815597 10.1080/14756366.2022.2086866 PMC9278437

[25] X. Liu , Y. Li , Q. Zhang , Q. Pan , P. Zheng , X. Dai , Z. Bai , W. D. Zhu , Front. Chem. 2022, 10, 815534. 35464202 10.3389/fchem.2022.815534 PMC9019572

[26] S. Pinheiro , E. M. C. Pinheiro , E. M. F. Muri , J. C. Pessôa , M. A. Cadorini , Greco , S.J. Biological Activities of [1,2,4]Triazolo[1,5‐a]pyrimidines and Analogs. Med. Chem. Res. 2020, 29, 1751.

[27] C. M. Richardson , D. S. Williamson , M. J. Parratt , J. Borgognoni , A. D. Cansfield , P. Dokurno , G. L. Francis , R. Howes , J. D. Moore , J. B. Murray , A. Robertson , A. E. Surgenor , C. J. Torrance , Bioorg. Med. Chem. Lett. 2006, 16, 1353. 16325401 10.1016/j.bmcl.2005.11.048

[28] U. Asghar , A. K. Witkiewicz , N. C. Turner , Knudsen , Nat. Rev. Drug Discov. 2015, 14, 130. 25633797 10.1038/nrd4504 PMC4480421

[29] C. Ballatore , K. Oukoloff , B. Lucero , K. R. Francisco , Brunden , K.R. 1,2,4‐Triazolo[1,5‐a]pyrimidines in Drug Design. Eur. J. Med. Chem. 2019, 165, 332. 10.1016/j.ejmech.2019.01.027 PMC6394845 30703745

[30] S. Pinheiro , E. M. C. Pinheiro , E. M. F. Muri , J. C. Pessôa , M. A. Cadorini , S. J. Greco , Med. Chem. Res. 2020, 29, 1751.

[31] Y. Deng , S. Chen , S. Wang , Q. Yu , B. Xu , L. Yang , Y. Zhou , H. Xie , Y. Liu , Y. Li , Bioorg. Med. Chem. Lett. 2011, 21, 692.

[32] J. Kovalevich , A. S. Cornec , K. Lou , G. Lewandowski , E. Somoza , J. Wang , P. Sharma , B. Keller , J. Burns , K. R. Brunden , et al, Neurobiol. Dis. 2016, 88, 106.

[33] B. Zhang , Q. Yu , Y. He , L. Yang , S. Chen , Y. Deng , B. Xu , J. Med. Chem. 2018, 61, 5784.

[34] E. McMillan , N. Wei , J. Carmody , A. N. Kang , S. Darensburg , T. Dodd , J. V. Oakley , J. Solowiej , L. Nguyen , S. T. M. Orr , P. Chen , E. Johnson , X. Yu , W. C. Diehl , G. M. Gallego , M. Jalaie , R. A. Ferre , S. Cho‐Schultz , H. Shen , J. G. Deal , Q. Zhang , T. R. Baffi , M. Xu , W. Roh , J. Lapira‐Miller , J. Goudeau , Y. Yu , R. Gupta , K. Kim , S. G. Dann , Z. Kan , J. C. Kath , S. K. Nair , N. Miller , B. W. Murray , A. R. Nager , C. Quinlan , M. D. Petroski , C. Zhang , A. Sacaan , T. VanArsdale , L. Anders , Cancer Cell 2025, 43, 464.40068598

[35] A. Shanabag , J. Armand , E. Son , H. W. Yang , Exp. Mol. Med. 2025, 57, 312. 39930131 10.1038/s12276-025-01395-3 PMC11873051

[36] C. Jiang , Y. Ye , W. Kang , J. Yang , Z. He , Q. Cao , C. Lian , Y. Xing , Q. Yang , J. Zhao , S. Pan , M. Feng , C. Song , Z. Liu , R. Wang , F. Yin , Y.‐D. Wu , J. Chen , Y. Huang , J. Med. Chem. 2025, 68, 1499. 39754579 10.1021/acs.jmedchem.4c01686

[37] M. S. Ibrahim , B. Farag , J. Y. Al‐Humaidi , M. E. Zaki , M. Fathalla , S. M. Gomha , Molecules 2023, 28, 3869. 37175279 10.3390/molecules28093869 PMC10180502

[38] G. R. Jang , R. Z. Harris , Lau , Med. Res. Rev. 2001, 21, 382. 11579439 10.1002/med.1015

[39] H. R. M. Rashdan , A. H. Abdelmonsef , I. A. Shehadi , S. M. Gomha , A. M. M. Soliman , H. K. Mahmoud , Molecules 2020, 25, 4997. 33126630 10.3390/molecules25214997 PMC7663531

[40] A. Alghamdi , A. S. Abouzied , A. Alamri , S. Anwar , M. Ansari , I. Khadra , Y. H. Zaki , S. M. Gomha , Curr. Issues Mol. Biol. 2023, 45, 1422. 36826038 10.3390/cimb45020093 PMC9955078

[41] I. M. Abbas , M. A. Abdallah , S. M. Gomha , M.S.H. Kazem , J. Heterocycl. Chem. 2017, 54, 3447.

[42] S. M. Gomha , H. M. Abdel‐Aziz , M. G. Badrey , Abdulla , J. Heterocycl. Chem. 2019, 56, 1275.

[43] S. M. Gomha , H. A. Abdel‐Aziz , Heterocycles 2012, 85, 2291.

[44] H. R. M. Rashdan , S. M. Gomha , M. S. El‐Gendey , M. A. El‐Hashash , A. M. M. Soliman , Green Chem. Lett. Rev. 2018, 11, 264.

[45] S. M. Gomha , A. O. Abdelhamid , O. M. Kandil , S. M. Kandeel , Abdelrehem , Mini‐Rev. Med. Chem. 2018, 18, 1670. 29692239 10.2174/1389557518666180424113819

[46] S. M. Gomha , F. M. Abdelrazek , A. H. Abdelrahman , P. Metz , J. Heterocycl. Chem. 2018, 55, 1729.

[47] H. H. Al‐Hujaj , A. A. Majed , Q. R. Abdalzahra , D. S. Abid , N. H. Faisal , H. H. Nameh , A. Naser , M. E. A. Zaki , S. A. Al‐Hussain , S. M. Gomha , A. Elhenawy , Abdellah , J. Mol. Struct. 2025, 1340, 142544.

[48] M. G. Badrey , A. A. Elhenawy , M. M. Alam , S. Nazreen , M. E. A. Zaki , S. M. Gomha , Res. Chem. 2025, 16, 102313.

[49] E. Irrou , Y. Ait Elmachkouri , S. El Haddad , B. K. Chagaleti , J. T. Magued , M. K. Kathiravan , S. A. Alotaibi , S. M. Gomha , A. Oubella , O. Hassan , N. K. Sebbar , Taha , J. Mol. Struct. 2025, 1338, 142310.

[50] P. Salehi , M. Dabiri , M. A. Zolfigol , M. Baghbanzadeh , Heterocycles 2005, 65, 1177.

[51] A. S. Shawali , S. M. Gomha , Regioselectivity in 1,5‐Electrocyclization of N‐[as‐Triazin‐3‐Yl]nitrilimines Tetrahedron 2002, 58, 8559.

[52] S. M. Gomha , S. M. Riyadh , Arkivoc 2009, xi, 58.

[53] S. M. Gomha , M. M. Edrees , F. M. A. Altalbawy , Int. J. Mol. Sci. 2016, 17, 1499. 27618013 10.3390/ijms17091499 PMC5037776

[54] S. Nagare , K. B. Lokhande , K. V. Swamy , J. Mol. Model. 2023, 29, 90. 36881272 10.1007/s00894-023-05496-6

[55] S. Kalra , G. Joshi , A. Munshi , R. Kumar , Eur. J. Med. Chem. 2017, 142, 424. 28911822 10.1016/j.ejmech.2017.08.071

[56] C. Bouclier , M. Simon , G. Laconde , M. Pellerano , S. Diot , S. Lantuejoul , B. Busser , L. Vanwonterghem , J. Vollaire , V. Josserand , Theranostics 2020, 10, 2008. 32104498 10.7150/thno.40971 PMC7019173

[57] W. Zhang , Y. Liu , H. Jang , R. Nussinov , JACS Au 2024, 4, 1911. 38818077 10.1021/jacsau.4c00138 PMC11134382

[58] S. M. Gomha , S. M. Riyadh , R. A. Alharbi , M. E. Zaki , T. Z. Abolibda , B. G. Farag , Cryst. Basel 2023, 13, 260.

[59] J. Clayton , A. Romany , E. Matenoglou , E. Gavathiotis , P. I. Poulikakos , Shen ,bioRxiv 2023. 10.7554/eLife.95334 PMC11825127 39945510

[60] P. Sekar , S. Arivanantham , P. Jaishanker , N. Sundhararajan , Y. Nagalingam , S. K. Raju , Res. Pharm. 2024, 14, 1.

[61] D. E. Pires , L. M. Kaminskas , D. B. Ascher ,in Computational Drug Discovery and Design, Humana Press, New York, NY, USA 2018, 271.

[62] S. A. Ouf , S. M. Gomha , B. Farag , M. E. Zaki , M. M. Ewies , I. A. Sharawy , F. O. Khalil , H. K. Mahmoud , Results Chem. 2024, 7, 101406.

[63] P. P.3D. Labute , Proteins Struct. Funct. Bioinf. 2009, 75, 187.

[64] T. Z. Abolibda , M. Fathalla , B. Farag , M. E. Zaki , S. M. Gomha , Molecules 2023, 28, 689. 36677750 10.3390/molecules28020689 PMC9861390

[65] S. M. Gomha , T. Z. Abolibda , A. H. Alruwaili , B. Farag , W. E. Boraie , S. A. Al‐Hussain , M. E. Zaki , A. M. Hussein , Catalysts 2024, 14, 489.

[66] D. E. Pires , T. L. Blundell , Ascher , J. Med. Chem. 2015, 58, 4066. 25860834 10.1021/acs.jmedchem.5b00104 PMC4434528

[67] R. S. Obach , F. Lombardo , N. J. T. Waters , Drug Metab. Dispos. 2008, 36, 1385. 18426954 10.1124/dmd.108.020479

[68] T. L. Moda , L. G. Torres , A. E. Carrara , A. D. Andricopulo ,Bioinformatics 2008, 24, 2270. 18684738 10.1093/bioinformatics/btn415

[69] D. Cao , J. Wang , R. Zhou , Y. Li , H. Yu , T. Hou , J. Chem. Inf. Model. 2012, 52, 1132. 22559792 10.1021/ci300112j

[70] F. Cheng , W. Li , Y. Zhou , J. Shen , Z. Wu , G. Liu , P. W. Lee , Y. Tang . J. Chem. Inf. Model. 2012, 52, 3099. 23092397 10.1021/ci300367a

[71] J. Ahmed , C. L. Worth , P. Thaben , C. Matzig , C. Blasse , M. Dunkel , R. Preissner , Nucleic Acids Res. 2011, 39, D1049–D1054. 20965964 10.1093/nar/gkq969 PMC3013803

[72] S. M. Gomha , S. M. Riyadh , E. A. Mahmmoud , Heterocycles, 2015, 91, 1227

