# Supplementary material for: Design, Synthesis, Cytotoxicity Assessment, and Molecular Docking of Novel Triazolopyrimidines as Potent Cyclin‐Dependent Kinase 4 Inhibitors
Source: ChemistryOpen. 2025 Nov 2;15(4):e202500324. doi: 10.1002/open.202500324 (PMC13052328; doi:10.1002/open.202500324)
Supplement: Supplementary file 1 — Supplementary Material [file OPEN-15-e202500324-s001.pdf]

Supplementary material file

Design, Synthesis, Cytotoxicity Assessment, and Molecular Docking of Novel Triazolopyrimidines as Potent CDK4 Inhibitors

Tariq Z. Abolibda[1], Sami A. Al-Hussain[2], Basant Farag[3], Mohamed El-Naggar[4], Magdi E. A. Zaki[2], Emad S. A. Alhazmi[5], Adel S. M. Almohammadi[5], Sobhi M. Gomha[1*]

[1]Department of Chemistry, Faculty of Science, Islamic University of Madinah, Madinah 42351, Saudi Arabia, t.z.a@iu.edu.sa (TA), smgomha@iu.edu.sa (SG).

[2]Department of Chemistry, Faculty of Science, Imam Mohammed Ibn Saud Islamic University (IMSIU), Riyadh 11623, Saudi Arabia, sahussain@imamu.edu.sa (SA), mezaki@imamu.edu.sa (MZ).

[3]Department of Chemistry, Faculty of Science, Zagazig University, Zagazig 44519, Egypt; basantfarag@zu.edu.eg.

[4]Depatement of Chemistry, Pure and Applied Chemistry Group, Faculty of Sciences, University of Sharjah, Sharjah 27272, UAE; melnagrr@sharjah.ac.ae.

[5]Medical Center, Islamic University of Madinah, Madinah 42351, Saudi Arabia, 800011@iu.edu.sa (EA), asalmohammadi@iu.edu.sa (AA).

* **Corresponding authors:** smgomha@iu.edu.sa

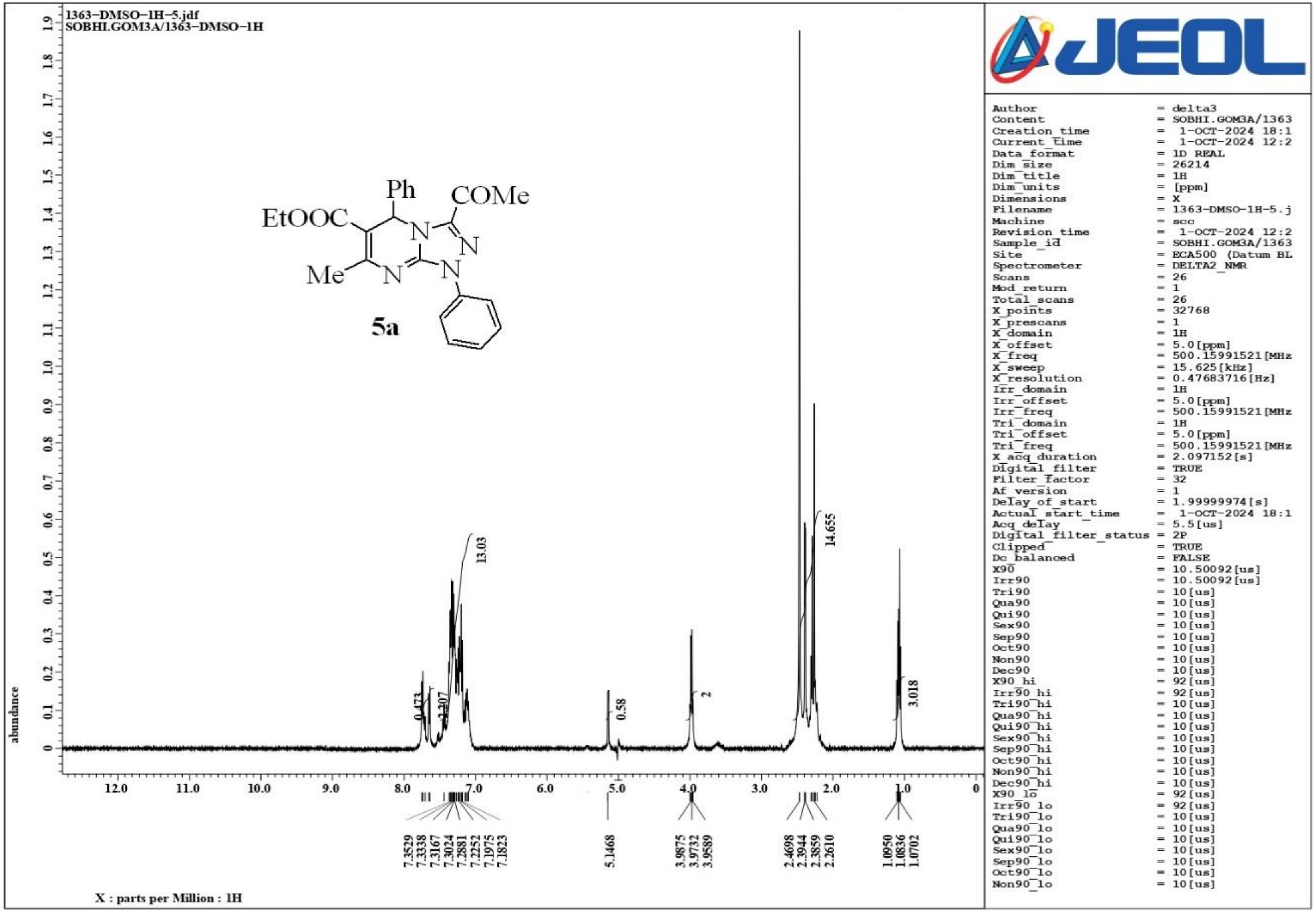

^{1}H-NMR spectra of compound **5a**

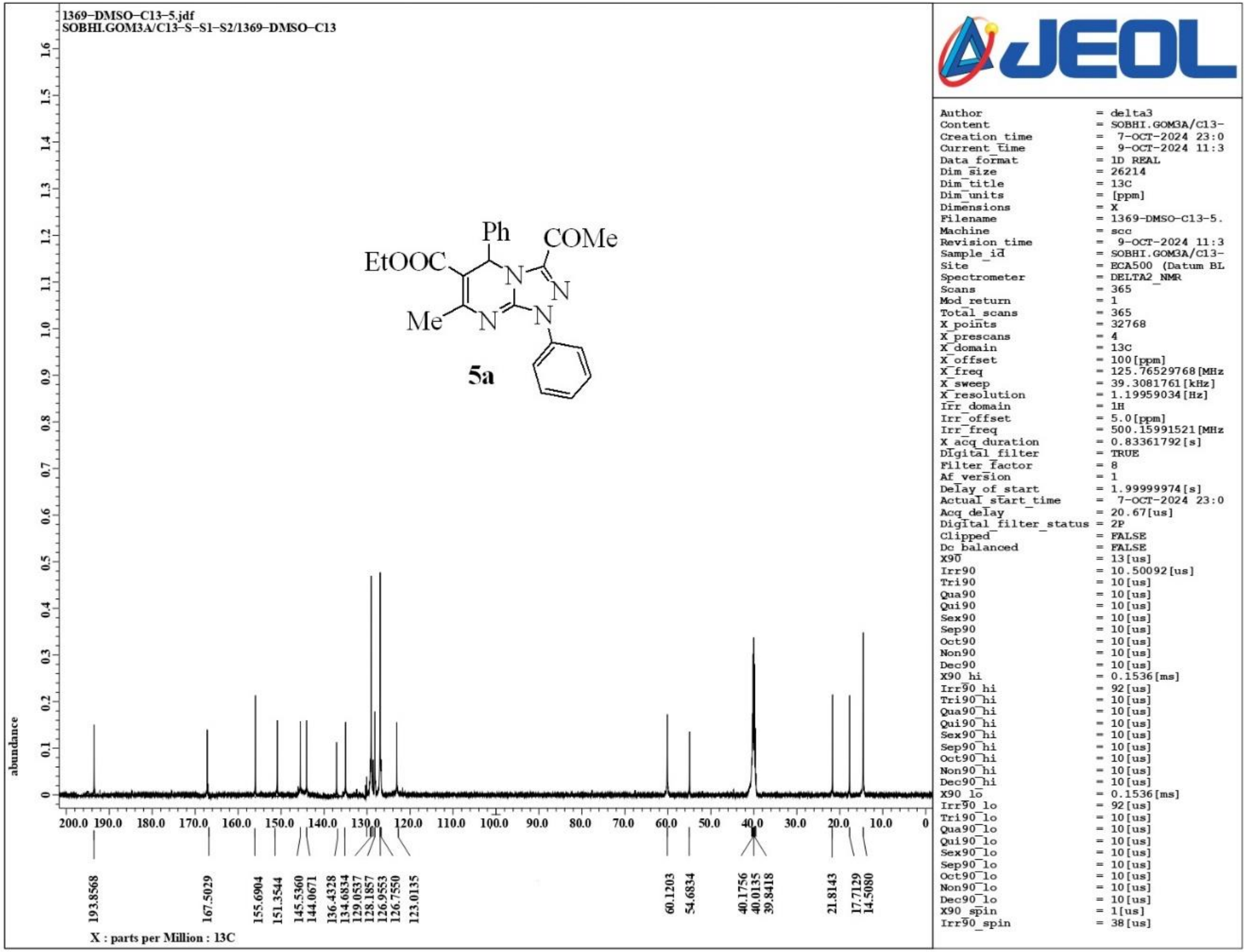

^{13}H-NMR spectra of compound **5a**

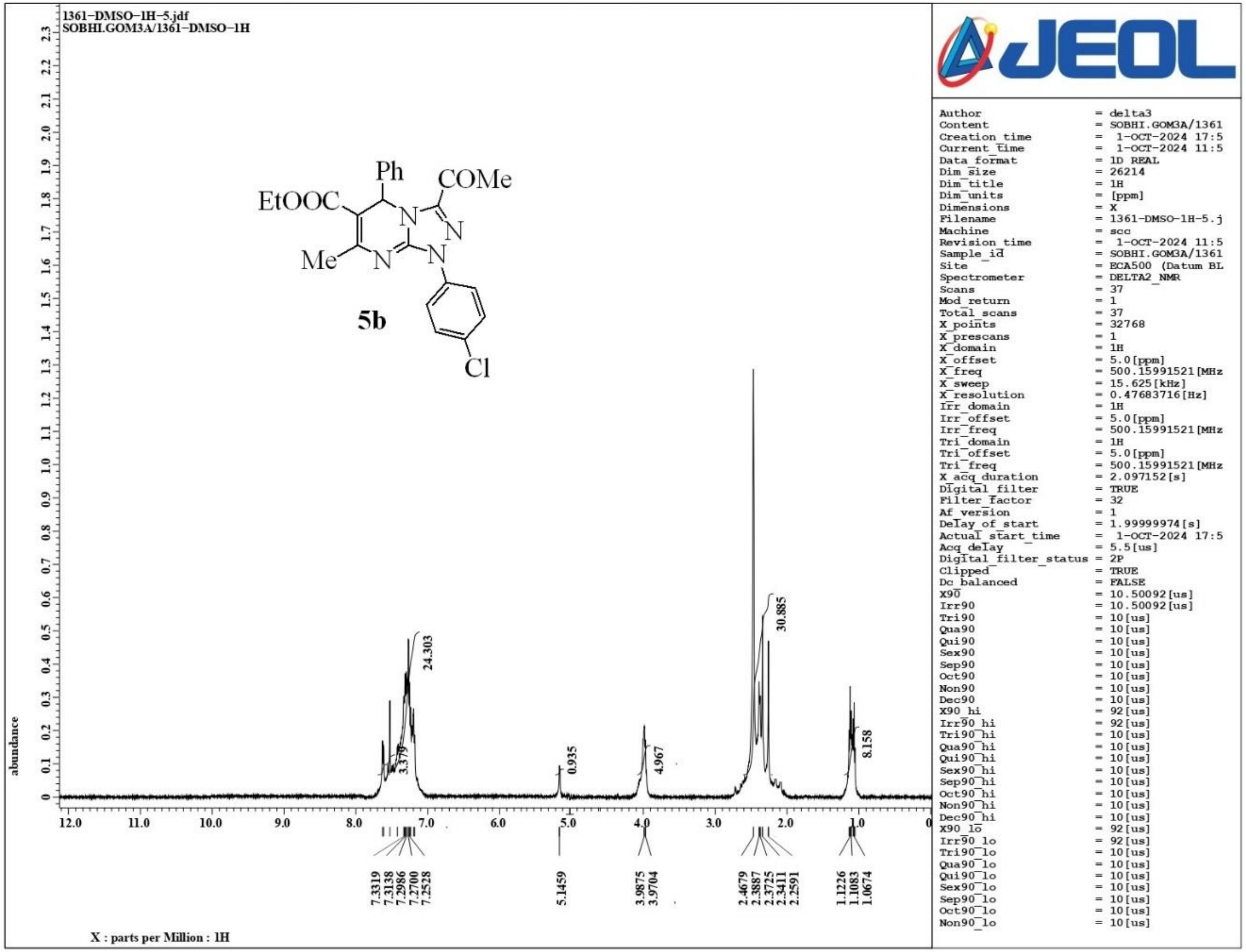

^{1}H-NMR spectra of compound **5b**

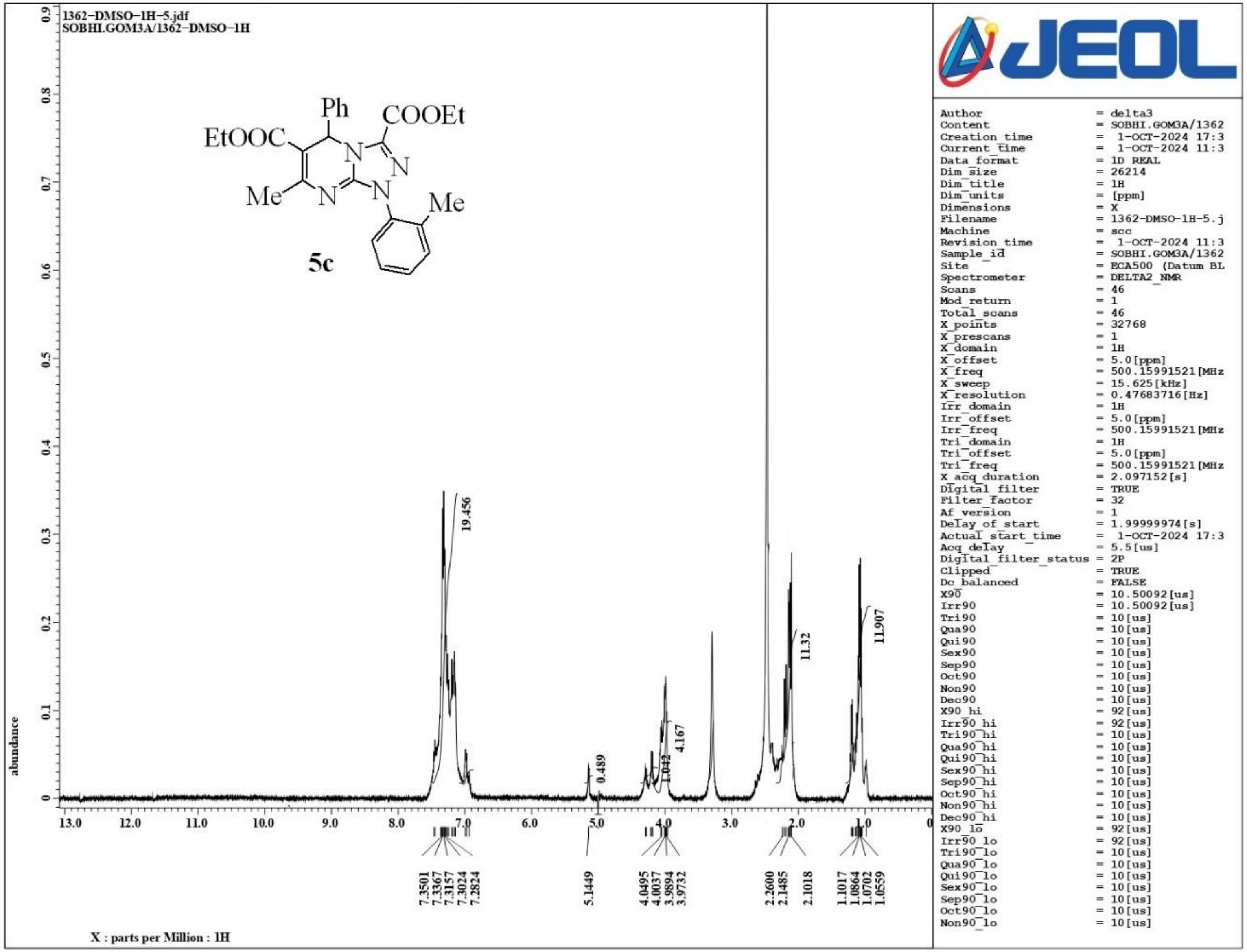

^{1}H-NMR spectra of compound **5c**

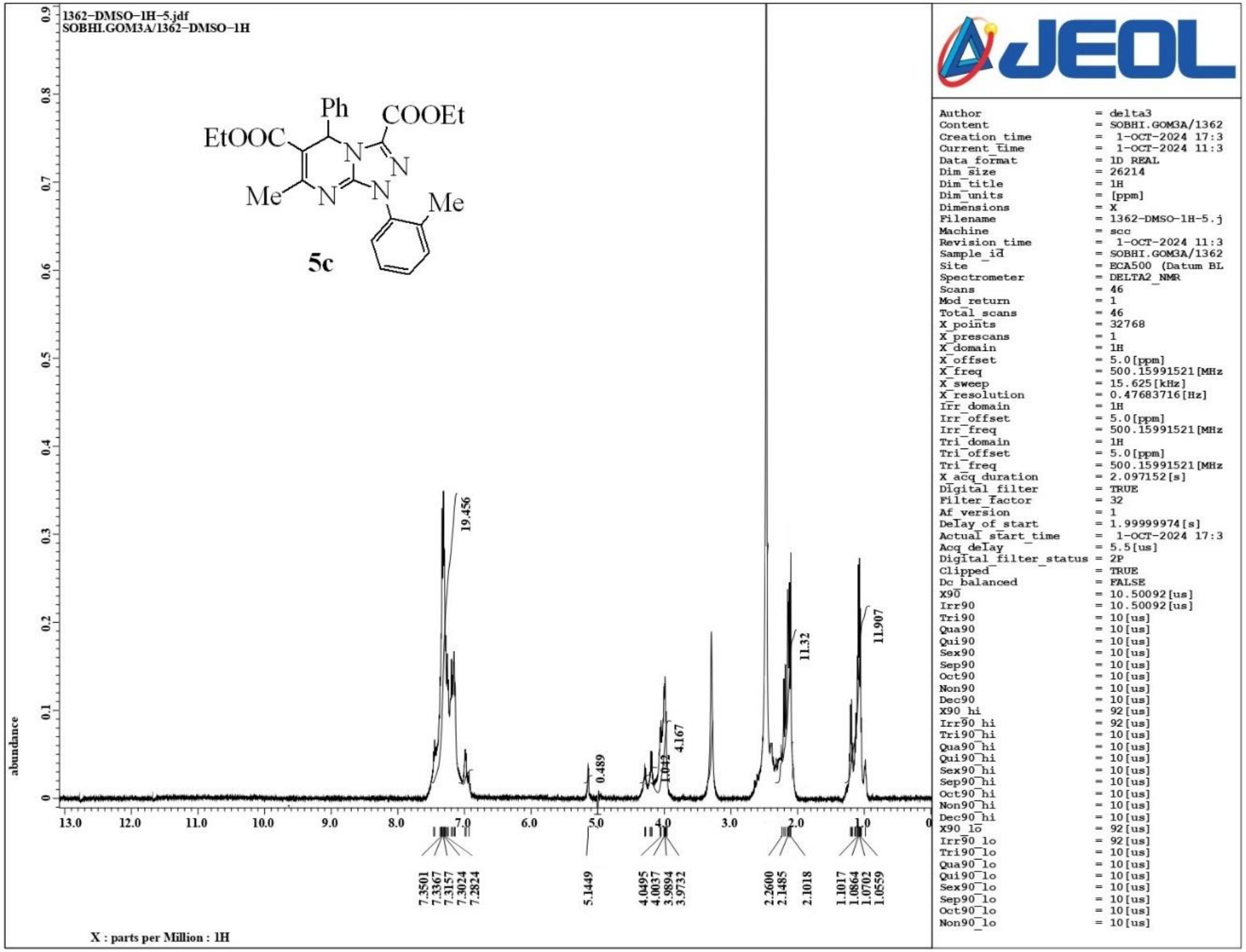

^{1}H-NMR spectra of compound **5e**

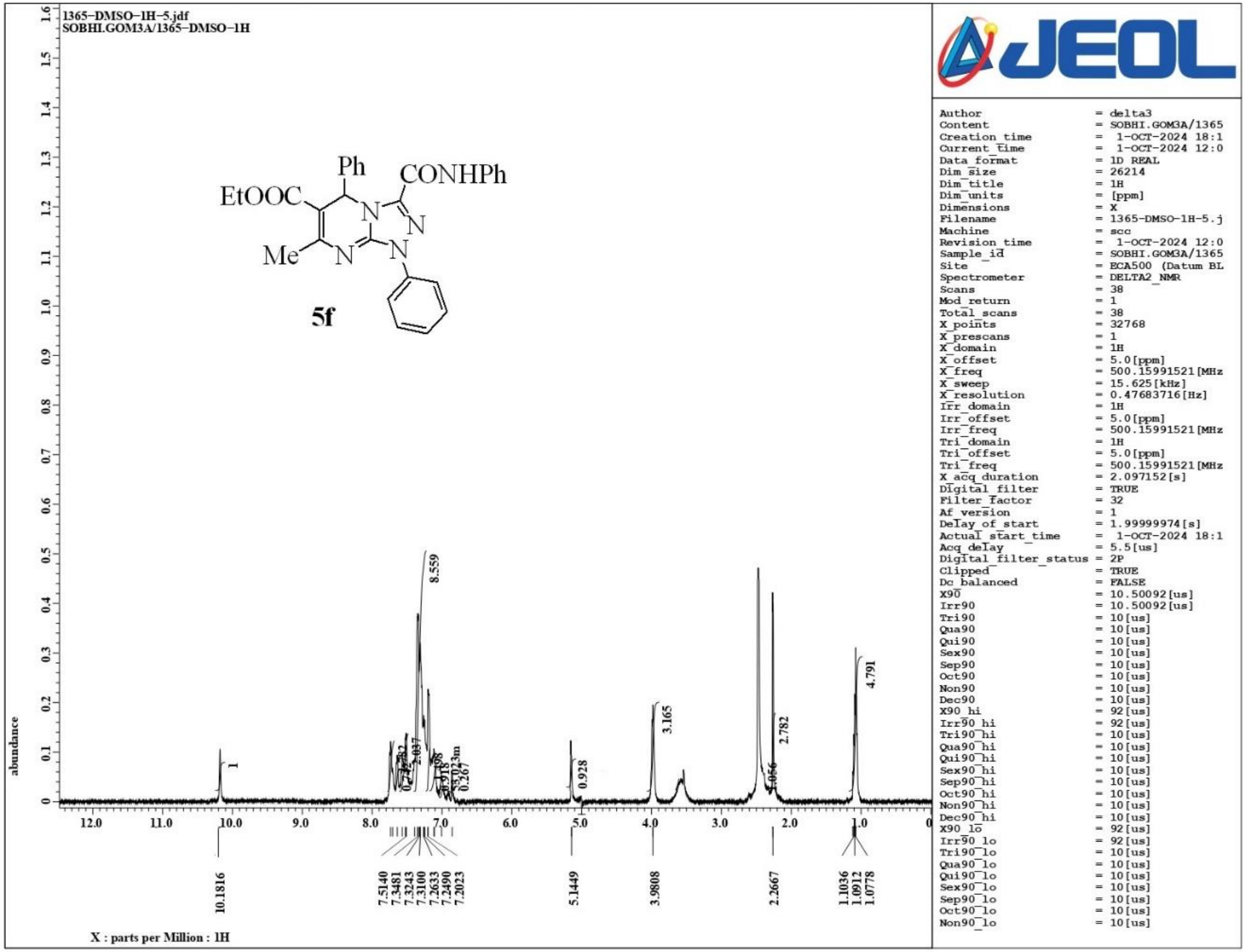

^{1}H-NMR spectra of compound **5f**

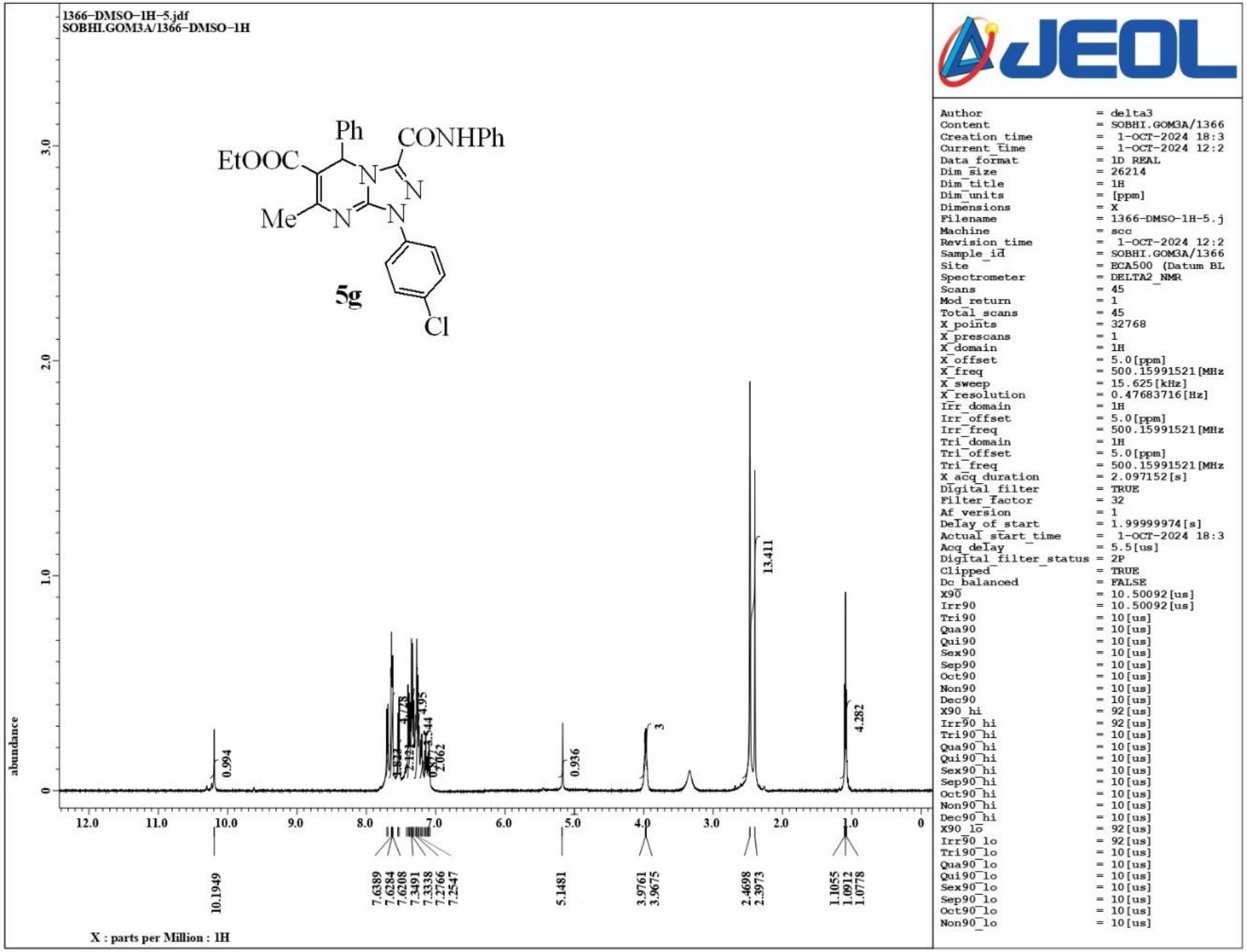

^{1}H-NMR spectra of compound **5g**

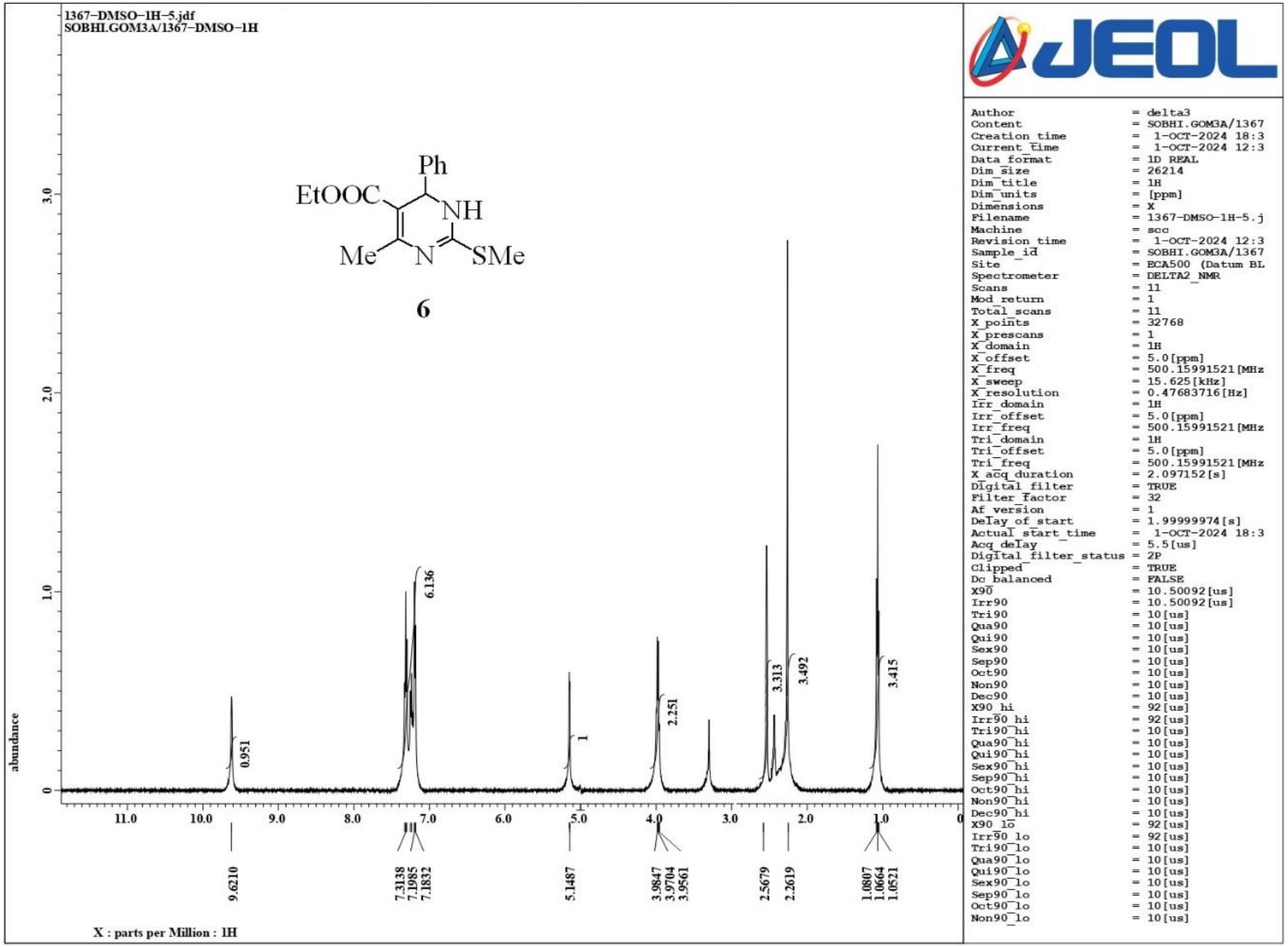

^{1}H-NMR spectra of compound **6**

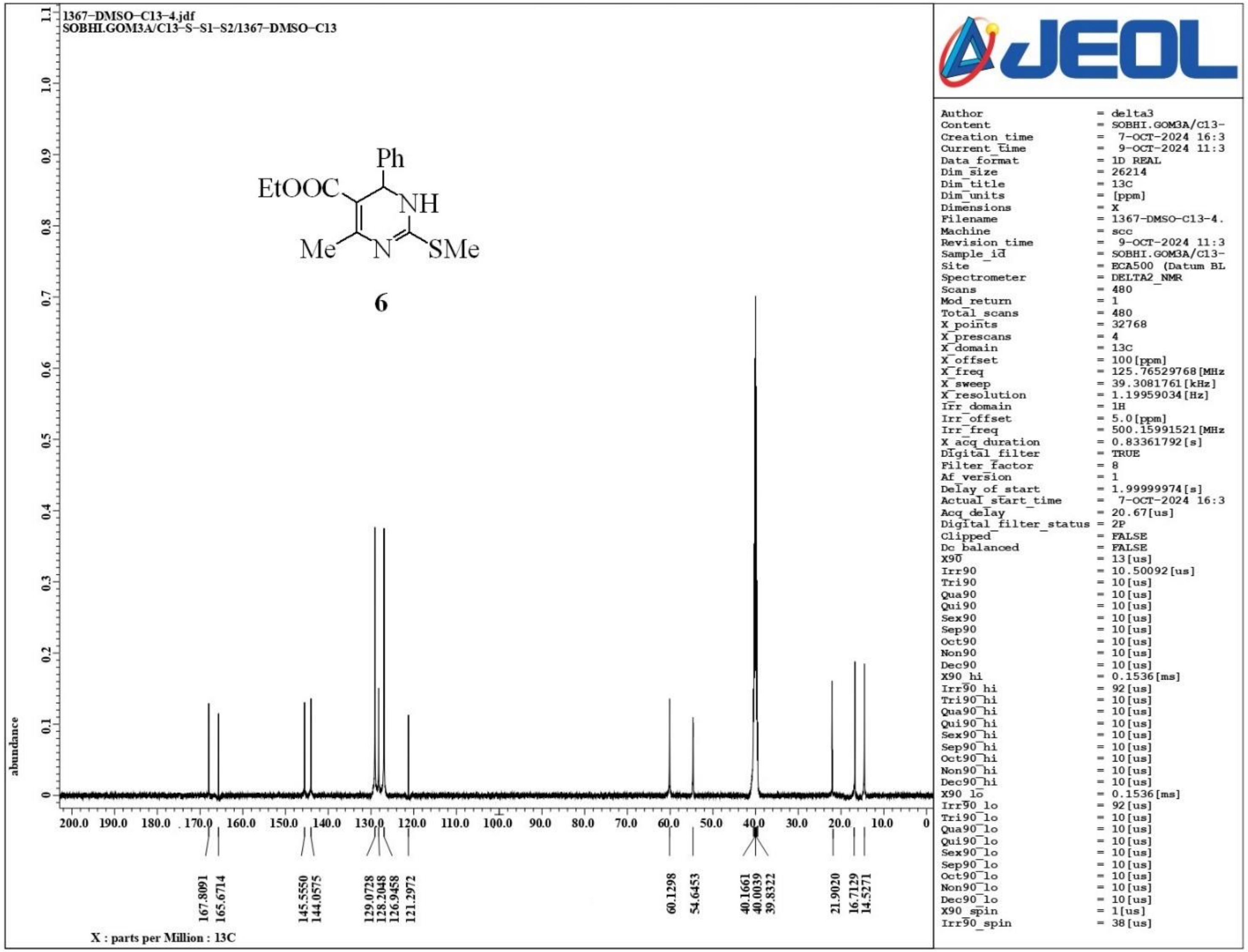

^{13}C-NMR spectra of compound **6**

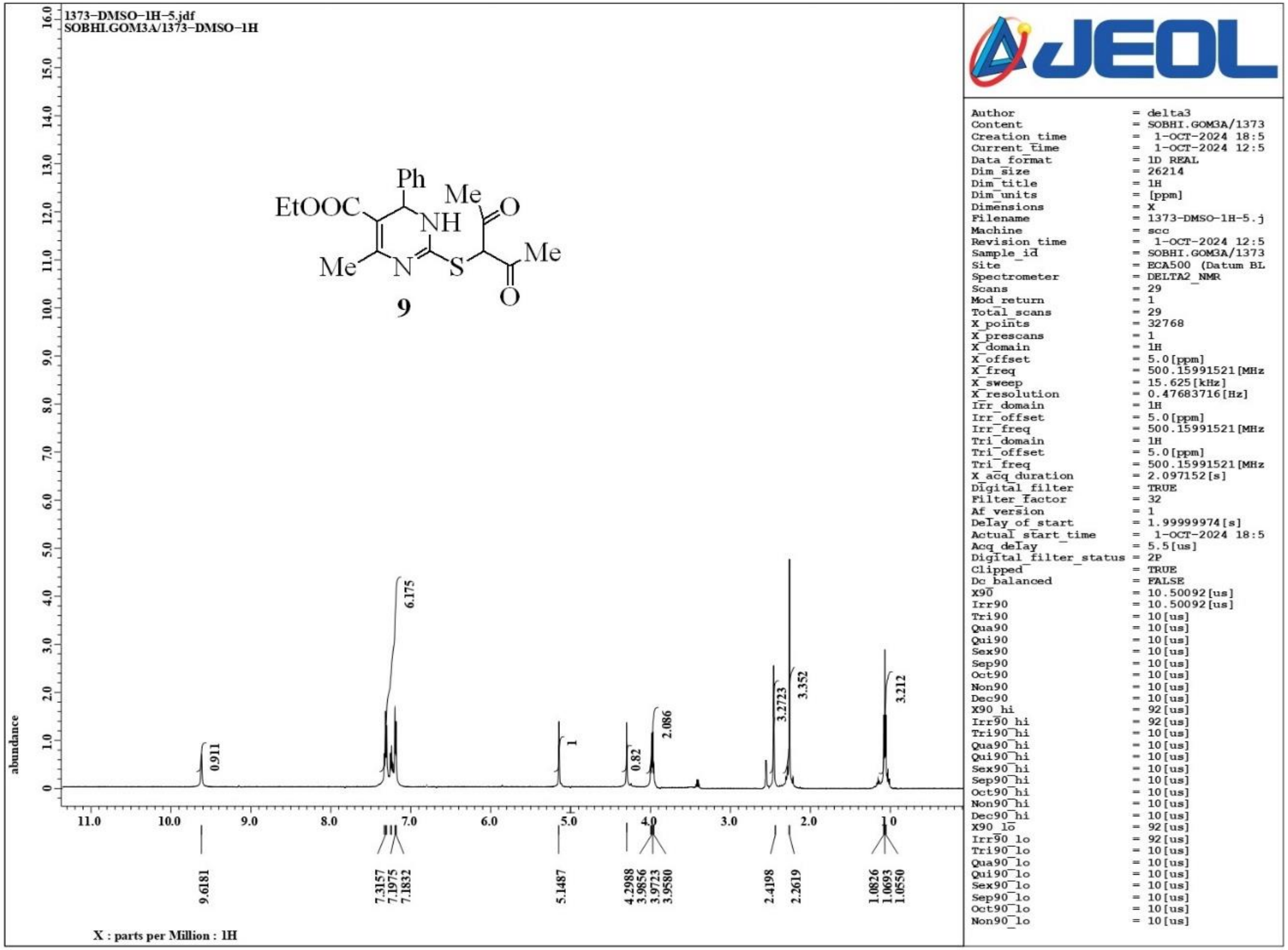

^{1}H-NMR spectra of compound **9**

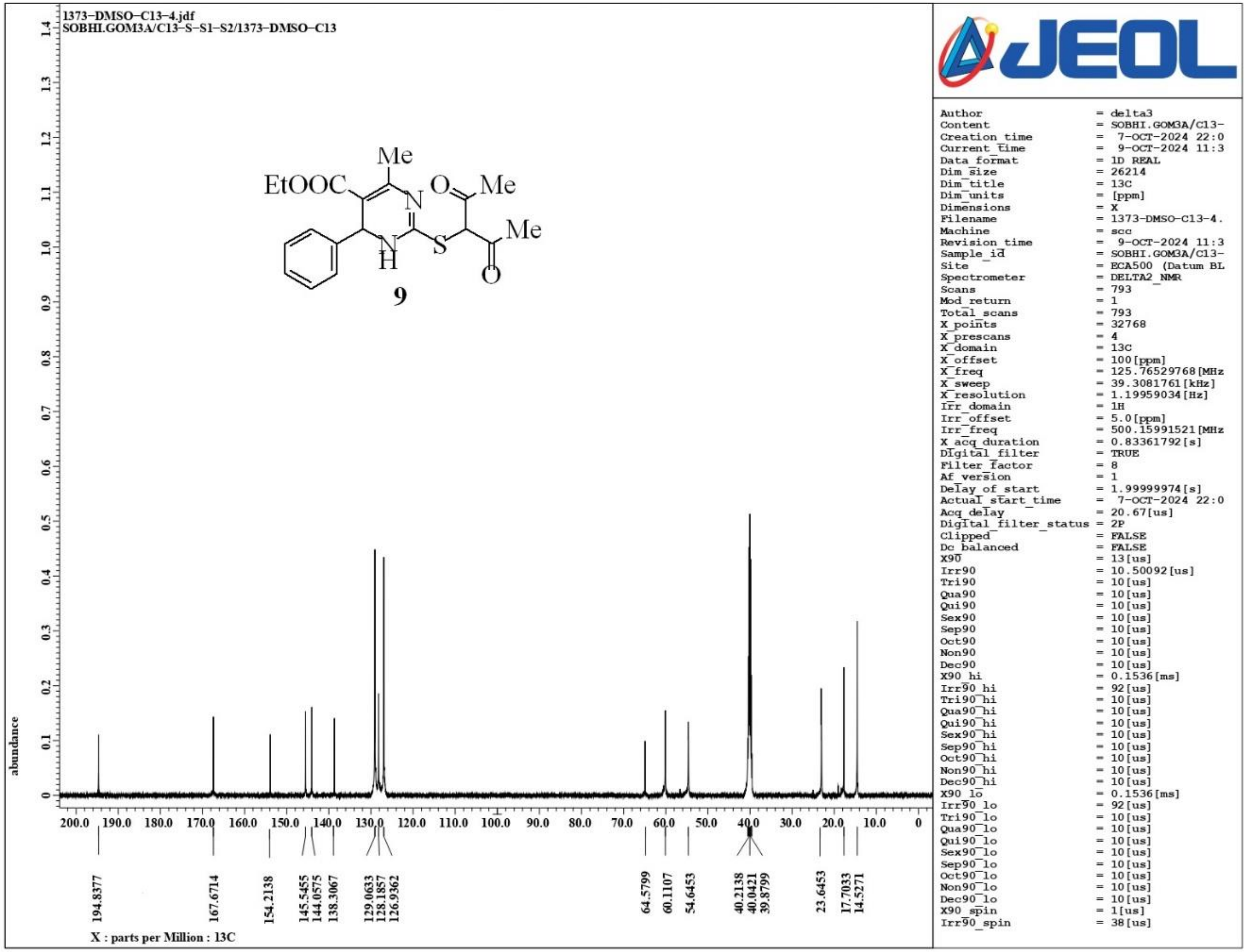

^{13}H-NMR spectra of compound **9**
